# Fibroblast‐specific palladin drives kidney fibrosis via MRTF–SRF signaling

**DOI:** 10.1002/path.6485

**Published:** 2025-10-24

**Authors:** Naoki Yamamoto, Norihiko Sakai, Yuta Yamamura, Daichi Kaikoi, Daiki Hayashi, Takahiro Matsuno, Akihiko Koshino, Keisuke Sako, Keisuke Horikoshi, Takahiro Yuasa, Akira Tamai, Taichiro Minami, Megumi Oshima, Shiori Nakagawa, Shinji Kitajima, Akinori Hara, Miho Shimizu, Jumpei Terakawa, Shin‐ichi Horike, Takiko Daikoku, Atsushi Mizokami, Hiroko Ikeda, Moeno Kadoguchi, Hiroshi Arakawa, Sumio Ohtsuki, David Lagares, Takashi Wada, Yasunori Iwata

**Affiliations:** ^1^ Department of Nephrology and Rheumatology Kanazawa University Kanazawa Japan; ^2^ Division of Blood Purification Kanazawa University Hospital Kanazawa Japan; ^3^ Graduate School of Veterinary Science Azabu University Sagamihara Japan; ^4^ Laboratory of Toxicology, School of Veterinary Medicine Azabu University Sagamihara Japan; ^5^ Division of Integrated Omics Research, Research Center for Experimental Modeling of Human Disease Kanazawa University Kanazawa Japan; ^6^ Division of Animal Disease Model, Research Center for Experimental Modeling of Human Disease Kanazawa University Kanazawa Japan; ^7^ Department of Integrative Cancer Therapy and Urology Kanazawa University Kanazawa Japan; ^8^ Department of Diagnostic Pathology Kanazawa University Hospital Kanazawa Japan; ^9^ Faculty of Pharmaceutical Sciences, Institute of Medical, Pharmaceutical and Health Sciences Kanazawa University Kanazawa Japan; ^10^ Department of Pharmaceutical Microbiology, Faculty of Life Sciences Kumamoto University Kumamoto Japan; ^11^ Fibrosis Research Center, Center for Immunology and Inflammatory Diseases, Division of Pulmonary and Critical Care Medicine Massachusetts General Hospital, Harvard Medical School Boston MA USA; ^12^ Zenon Biotech Boston MA USA

**Keywords:** CKD, fibrosis, fibroblast, palladin, MRTF, SRF, TGF‐β1, actin cytoskeleton, actin‐associated protein, proteome analysis

## Abstract

Fibrosis is a common end‐stage pathway of progressive chronic kidney diseases. Previously we demonstrated that myocardin‐related transcription factor (MRTF)–serum response factor (SRF) signaling drives the expression of fibrosis‐related molecules through actin cytoskeleton dynamics in renal fibroblasts. However, it has not been elucidated whether actin‐associated proteins relate to the pathogenesis of fibrosis. Here, we reveal that the actin cytoskeleton‐regulating pathway is significantly correlated with estimated glomerular filtration rate (eGFR) and collagen type 1 alpha 1 expression in human proteome analysis. We found that palladin was one of the TGF‐β1‐dependent actin‐associated proteins in renal fibroblasts. Our mechanistic studies demonstrated that palladin activates MRTF–SRF signaling via actin cytoskeleton rearrangement upon TGF‐β1 stimulation. In addition, palladin expression itself was enhanced by MRTF–SRF signaling, indicating a positive feedback loop. *In vitro*, genetic silencing of the palladin–MRTF–SRF axis suppressed extracellular matrix production and myofibroblast differentiation. In preclinical models *in vivo*, fibroblast‐specific palladin‐deficient mice (palladin^iFBKO^) were protected from kidney dysfunction and fibrosis that developed in adenine‐induced nephropathy, which was associated with reduced numbers of myofibroblasts compared to wild type (palladin^F/F^) mice. In patients with renal disease, palladin was significantly upregulated in the renal interstitium of patients with low eGFR and kidney fibrosis. Moreover, upregulation of the palladin–MRTF–SRF axis correlated with kidney function and fibrosis in patients with various kidney diseases, including IgA nephropathy, diabetic nephropathy, and nephrosclerosis. Taken together, we consider palladin to be a novel regulator of actin cytoskeleton signaling in fibrotic fibroblasts and represents a novel therapeutic target for the treatment of progressive kidney diseases. © 2025 The Author(s). *The Journal of Pathology* published by John Wiley & Sons Ltd on behalf of The Pathological Society of Great Britain and Ireland.

## Introduction

Chronic kidney disease (CKD) is a progressive condition resulting in end‐stage kidney disease that requires renal replacement therapy. The high prevalence of CKD gives rise to other organ dysfunction with a high mortality rate [1, 2]. Regardless of the primary disease, the common pathological feature of progressive CKD has been shown to be fibrosis, which is also responsible for various other organ failures [3, 4]. Pathogenesis of fibrosis involves aberrant wound‐healing processes, characterized by extracellular matrix (ECM) deposition and accumulation of fibroblasts/myofibroblasts [5, 6]. In this fibrotic process, self‐perpetuating damage is maintained through a positive feedback loop between fibrotic stimuli such as transforming growth factor (TGF)‐β1 and its responses [7, 8]. Elucidation of precise mechanisms of how aberrant wound‐healing processes are formed leads to a novel treatment for CKD.

There has been growing interest in understanding actin cytoskeleton‐dependent signaling as a driver of embryogenesis, wound healing, fibrosis, cancer metastasis, and inflammation [9]. It is now well recognized that the actin cytoskeleton is dynamically organized via the assembly of globular actin (G‐actin) into filamentous actin (F‐actin) and their disassembly. Various actin‐associated proteins regulate the actin filament assembly and disassembly as well as a change in filament conformation, playing a wide variety of roles, such as cross‐linking, scaffolding, and severing [10]. Stabilization of these actin filaments is mostly regulated by focal adhesion proteins such as vinculin, which link the actin cytoskeleton with the ECM via integrins [11, 12]. Cofilin and profilin are involved in the process of elongation and severing of the actin filament [13, 14, 15, 16]. More recently, palladin has emerged as a novel scaffold protein that regulates intracellular force generation in cells by the cross‐linking of actin filaments [17, 18]. However, it has yet to be understood whether actin‐associated proteins relate to the pathogenesis of fibrosis.

A growing body of evidence suggests that focal adhesions and actin cytoskeleton signaling regulate multiple cellular functions, including cell morphology, division, migration, and differentiation [9, 19]. More importantly, focal adhesions and the actin cytoskeleton act as cellular sensors of force, controlling mechanotransduction pathways activated by external cues such as matrix stiffness or intracellular tension induced by TGF‐β1 [6, 20, 21]. In this regard, we previously demonstrated that myocardin‐related transcription factors (MRTFs) are involved in the expression of focal adhesion‐related molecules such as vinculin and integrins through actin cytoskeleton reorganization [22, 23, 24]. MRTFs comprising MRTF‐A and MRTF‐B isoforms are transcriptional co‐activators for serum response factor (SRF). Polymerization of G‐actin into F‐actin releases MRTFs from G‐actin, resulting in the translocation of MRTFs to the nucleus, where they enhance SRF transcriptional activity [25, 26]. Previous studies have shown that MRTF–SRF signaling contributes to fibrosis in the lung, kidney, and peritoneum by driving both epithelial‐to‐myofibroblast and fibroblast‐to‐myofibroblast transitions [22, 24, 27, 28, 29]. While actin polymerization is proposed as a mechanism to trigger MRTF–SRF signaling, actin regulators of this pathway and their involvement with kidney fibrosis remain poorly understood.

Here, we analyzed proteomes and discovered that actin‐associated proteins were significantly correlated with kidney function and ECM expression. Of these, we further revealed that palladin, a TGF‐β1‐dependent target protein, promoted MRTF–SRF signaling via the formation of F‐actin, in turn enhancing the expression of palladin itself, forming a positive feedback loop between palladin and MRTF–SRF signaling in renal fibroblasts. Mechanistically, genetic ablation of palladin blocked TGF‐β1‐induced myofibroblast formation by targeting MRTF–SRF signaling. In *in vivo* study, fibroblast‐specific deletion of palladin in mice showed protection from kidney dysfunction and fibrosis in mouse models of CKD. In patients with renal disease, palladin was significantly upregulated in the renal interstitium of patients with low eGFR and correlated with kidney function and fibrosis in various kidney fibrotic diseases.

## Materials and methods

### Study approval

This study was approved by the medical ethics committee of Kanazawa University [registration number: 2016‐422 (846)] and was conducted in accordance with the Declaration of Helsinki. All participants provided written informed consent and were informed regarding their right to withdraw from the study at any time. All animal experiments were approved by the Institute for Experimental Animals, Kanazawa University Advanced Research Center (registration number: AP‐204115 and AP‐204127) and performed following the institutional guidelines.

### Reagents and cell culture experiments

Mouse primary renal fibroblasts were purchased from Cell Biologics (Chicago, IL, USA). Renal fibroblasts were cultured at 37 °C with 5% CO_2_ and grown in Dulbecco's Modified Eagle's Medium (DMEM) (Nacalai Tesque, Kyoto, Japan) with 10% fetal bovine serum (FBS) (Thermo Fisher Scientific, Waltham, MA, USA), 1% sodium pyruvate (Wako, Osaka, Japan), 1% nonessential amino acid mixture (Wako), 1% penicillin/streptomycin stock solution (Lonza, Walkersville, MD, USA), and 1% L‐glutamine (CellGenix, Portsmouth, NH, USA). Following overnight 16‐h serum deprivation, cells were stimulated with TGF‐β1 (R&D Systems, Minneapolis, MN, USA). CCG‐1423 (Ann Arbor, MI, USA) and latrunculin B (Thermo Fisher Scientific) were dissolved in DMSO (Sigma Aldrich, St. Louis, MO, USA) and pretreated 30 min before TGF‐β1 stimulation. The characteristics of renal fibroblasts were verified by immunofluorescence with fibroblast marker, fibroblast‐specific protein 1 and epithelial markers, E‐cadherin and zonula occludens‐1 (data not shown). Cells around passage 5 to 10 were used in *in vitro* experiments. In each experiment, renal fibroblasts were seeded at a density of 1.0 × 10^5^ cells/ml in 12‐well plates. Cells were analyzed or passaged before confluence to avoid the effects of cell density. We monitored routinely for mycoplasma contamination.

### Proteome analysis

Human kidney samples for proteome analysis were randomly collected after confirmation of negative surgical margins by urologists and pathologists in the resected kidneys. The influence of renal cell carcinoma on kidney function was preoperatively evaluated in a renogram. One patient on dialysis could not have renogram examination because of anuria. Proteins were extracted from formalin‐fixed paraffin‐embedded sections and digested using the SP3 method as described previously [30, 31]. The digested peptide samples were analyzed by sequential window acquisition of all theoretical mass spectra (SWATH‐MS) on a 6,600 TripleTOF instrument (SCIEX, Framingham, MA, USA) interfaced to an Eksigent NanoLC400 system (SCIEX) under the conditions reported previously [32]. Protein identification and quantification were performed using the library‐free search function DIA‐NN 1.9.2, with the UniProt human reference proteome allowing one miscleavage [33]. The intensities of the precursors were normalized using retention time‐dependent cross‐run normalization. Peptides and proteins were filtered at a false discovery rate of < 1% for identification and quantification.

### Animals

C57BL/6J mice were purchased from the Jackson Laboratory Japan, Inc. (Kanagawa, Japan). We utilized CRISPR/Cas9‐mediated targeting strategy to generate palladin‐floxed mice. The crRNAs targeting intron 11 (5′‐GGAAGGTTCGGACACCCATA‐3′) and intron 12 (5′‐GTCTCTCCTACTCACTAAGA‐3′) of the mouse *Palld* gene were designed using the online sgRNA design tool available at https://sg.idtdna.com/site/order/designtool/index/CRISPR_CUSTOM and purchased from Integrated DNA Technologies (Coralville, IA, USA). Donor single‐stranded DNA (ssDNA) was produced using the Guide‐it™ Long ssDNA Production System v2 (TAKARA BIO, Kusatsu, Japan), with IDT‐synthesized gBlocks Gene Fragments as templates. Fertilized pronuclear‐stage embryos were prepared by *in vivo* fertilization. Next, the ssODN (40 ng/μl) and the complex of crRNAs (0.31 μm) and tracrRNA (0.31 μm) and Cas9 protein (0.18 μm) (Integrated DNA Technologies) in Opti‐MEM (Thermo Fisher Scientific) were introduced to pronuclear‐stage embryos by microinjection. After injection, embryos were washed and cultured in a potassium simplex optimization medium (KSOM; ARK Resource) overnight. One hundred twenty‐three two‐cell embryos were transferred to pseudo‐pregnant recipient ICR mice (CLEA Japan, Inc., Tokyo, Japan).

Pups from transplanted embryos were considered the F0 generation. The genotypes of the F0 generation were analyzed by PCR with genomic DNA extracted from the tail and primers (supplementary material, Figure S1A). The PCR steps included an initial denaturation at 95 °C for 2 min followed by 33 cycles of 95 °C (30 s), 61 °C (30 s), and 72 °C (30 s) and a final extension at 72 °C (7 min). PCR products were electrophoresed on a 2% agarose gel, and DNA was visualized by ethidium bromide staining. To validate the CRISPR editing by Sanger sequencing, PCR was conducted using the following primers: forward primer 5′‐TGCCATCAGTACTTCCGTGT‐3′ and reverse primer 5′‐GCCACTGAATATGCTCCCCT‐3′. The resulting PCR products were cloned into the pGEM‐T Easy vector (Promega) by TA cloning, and Sanger sequencing was performed to determine the DNA sequence of the edited mice. Among seven founder (F0) mice obtained, one male mouse had a heterozygous KI allele. This male mouse was utilized for *in vitro* fertilization with wild type female eggs to obtain an F1 generation. Mice with tamoxifen‐inducible Cre‐recombinase under the control of *Col1a2* expression [Col1a2‐Cre‐ER(T)] were purchased from the Jackson Laboratory and analyzed by PCR with genomic DNA extracted from the tail and primers (supplementary material, Figure S1B). By crossing these mice, we generated inducible fibroblast‐specific palladin conditional knockout mice (palladin^iFBKO^). Mice without Cre‐recombinase expression were used as controls (palladin^F/F^). Tamoxifen (Sigma Aldrich) (100 mg/kg/day intraperitoneally for 5 days) was administered 2 weeks before adenine administration to induce palladin deletion. The efficiency of palladin suppression on fibroblasts was evaluated using dermal fibroblasts from mice 2 weeks after tamoxifen treatment (supplementary material, Figure S1C). All experiments used male and weight‐matched mice maintained in specific pathogen‐free environments at 8–10 weeks of age. Mice were assigned randomly to groups based on Mendelian inheritance.

### Kidney fibrosis model

Kidney fibrosis models using adenine and folic acid administration to mice were produced as described previously with a minor modification (supplementary material, Figure S1C) [34, 35]. In brief, adenine (Sigma Aldrich) was administered to mice at 50 mg/kg body weight in 0.5% carboxymethyl cellulose (CMC) (Wako) by oral gavage (Natume, Tokyo, Japan) 5 days a week for 4 weeks. Mice were sacrificed on day 28. Control mice received equal volumes of 0.5% CMC alone (vehicle) on the same schedule. Folic acid (Wako) was dissolved in 300 mm sodium bicarbonate (NaHCO_3_) (Wako) and injected intraperitoneally at a dose of 250 mg/kg body weight once weekly for 3 weeks. Mice were sacrificed on day 21. Control mice received equal volumes of 300 mM NaHCO_3_ on the same schedule. Time and order of administration were randomized in both experiments. The investigators could not be blinded to whether the animal was injected with vehicle, adenine, or folic acid due to the difference in color.

### NephroSeq database analyses

Data were obtained from the NephroSeq database, version 5 [36]. For selected genes (*PALLD*, *MKL1*, *MKL2*, *SRF*, and *COL1A1*), all tubulointerstitial expression data were downloaded for GFR analysis and interstitial fibrosis and tubular atrophy analysis. The data were filtered for *p* < 0.05 and Pearson's correlation coefficient for |*r*| > 0.5.

### Statistical analysis

All analyses were performed using GraphPad Prism 9.0 (GraphPad Software, San Diego, CA, USA). Data are expressed as mean ± SEM. Unpaired *t*‐tests were used to assess the significance of comparisons between two groups, and analysis of variance with *post hoc* Dunnett's multiple comparisons test was used for comparisons among more than two groups. *P* values of less than 0.05 were considered statistically significant for all comparisons.

Detailed methods are provided in Supplementary materials and methods including primers (supplementary material, Table S1) and antibodies (supplementary material, Table S2).

## Results

### Palladin, a TGF‐β1‐dependent actin‐associated protein, is upregulated in patients with kidney fibrosis and low eGFR

**Figure 1:**
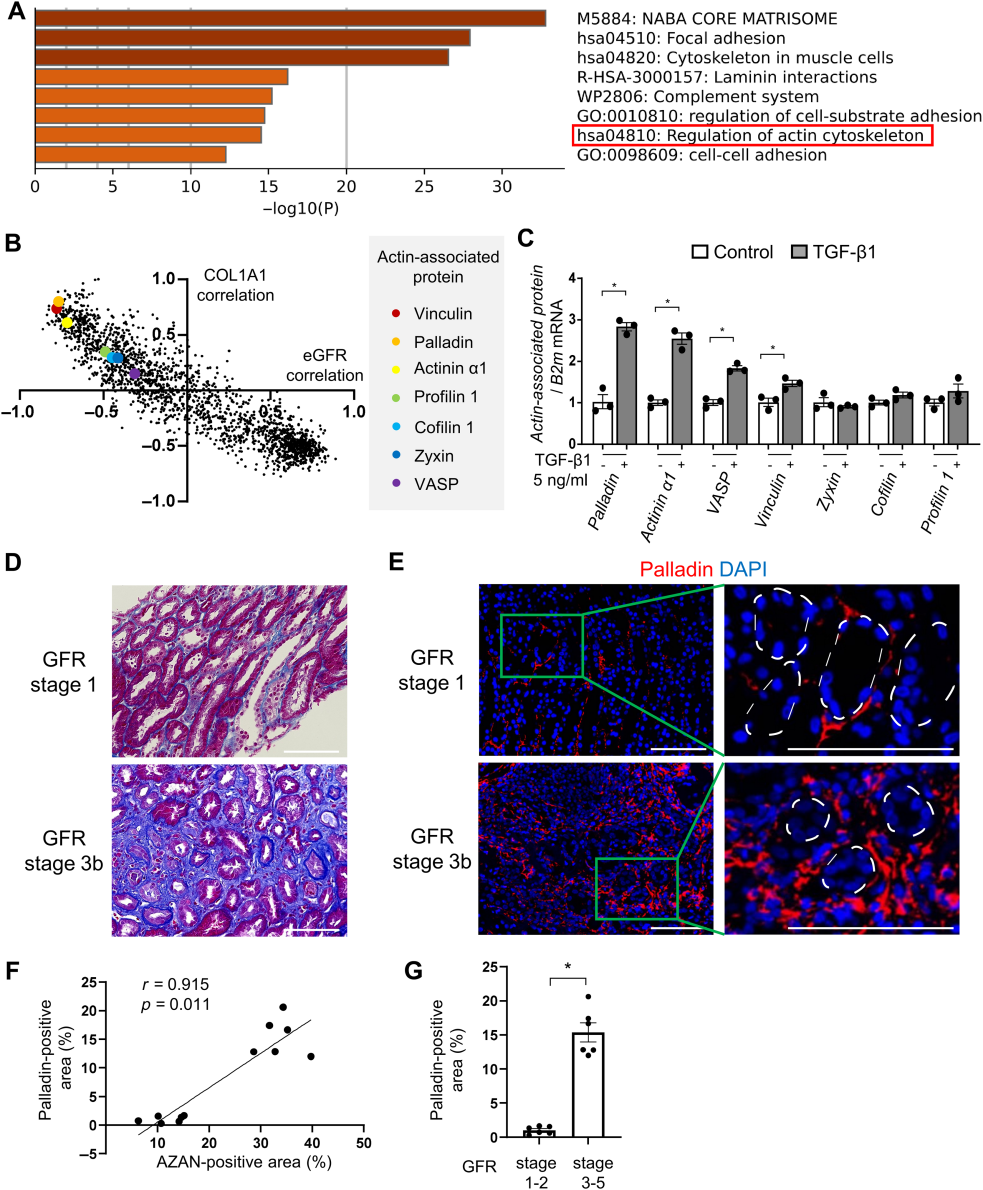
Palladin, a TGF‐β1‐dependent actin‐associated protein, is upregulated in patients with kidney fibrosis and low eGFR. (A) Enrichment analysis of proteins exhibiting Pearson's correlation coefficient for *r* < −0.7 with eGFR. (B) Correlation of each protein with eGFR and COL1A1. (C) Gene expressions of actin‐associated proteins induced by TGF‐β1 stimulation for 6 h (*n* = 3 cell preparations/group). (D and E) Representative kidney sections of (D) Azan‐Mallory and (E) palladin staining in patients with GFR stage 1 and GFR Stage 3b. White dotted lines show tubule borders. (F) Scatter plot revealing correlation between AZAN‐positive area and palladin‐positive area. (G) Quantitative analysis of palladin staining in kidney sections (*n* = 6 patients/group). Scale bars, 100 μm. Mean ± SEM. *Statistically significant.

To extract the molecules that correlate with kidney function and fibrosis, we performed proteome analysis of noncancerous lesions of renal cell carcinoma (RCC) in 16 patients (supplementary material, Figure S2A). Preoperative renogram revealed no significant difference in GFR between the tumor‐bearing and contralateral kidneys, indicating that RCC did not affect kidney function (supplementary material, Figure S2B). Proteins exhibiting Pearson's correlation coefficient of *r* < −0.7 with eGFR were identified for downstream enrichment analysis. Metascape analysis revealed significant enrichment of the actin cytoskeleton‐regulating pathway, suggesting its potential involvement with kidney dysfunction (Figure 1A). In addition, plotting the correlation coefficients for each protein in a two‐dimensional matrix demonstrated that some actin‐associated proteins were strongly related to kidney function and collagen type 1 alpha 1 chain (COL1A1), a major component of ECM (Figure 1B). We next evaluated the expression of these actin‐associated proteins by stimulating cultured murine renal fibroblasts with TGF‐β1, which drives fibrosis in multiple organs via upregulation of canonical SMAD signaling and noncanonical cytoskeletal‐dependent pathways [26, 37, 38]. As shown in Figure 1C, palladin, actinin α1, vasodilator‐stimulated phosphoprotein (VASP), and vinculin were upregulated by TGF‐β1. Focal adhesion‐related proteins are known to be involved in myofibroblast activation [39, 40]. In addition, it has been indicated that palladin's expression increases upon myofibroblast induction and precedes α‐smooth muscle actin (αSMA) upregulation [41, 42]. At the molecular level, palladin enhances the stabilization of F‐actin through binding to and cross‐linking actin filaments as well as binding to other focal adhesion proteins [17, 18, 43].

Previous studies showed that palladin was upregulated in the stroma of multiple tumors, including pancreas, lung, colon, and gastric cancer [44, 45], controlling cancer‐associated fibroblast activation and inducing invasive motility in metastatic cells [46, 47]. Although the presence of palladin has been reported in injured tubules [48], the role of palladin in human fibrotic diseases has not been studied yet. We therefore investigated palladin localization in kidney fibrosis. As shown in supplementary material, Figure S3A,B, palladin was detected intracellularly in interstitial cells and colocalized with αSMA‐expressing cells. In addition, the palladin‐positive area was positively correlated to the fibrotic area in Azan‐Mallory staining (Figure 1D–F). The proportion of palladin‐positive area was significantly higher in patients with GFR stage 3–5 than those with GFR stage 1–2 (Figure 1G). Collectively, our data indicate that palladin is a target gene of TGF‐β1 in renal fibroblasts and its expression correlated with the extent of kidney fibrosis in patients.

### Palladin controls actin cytoskeleton and MRTF–SRF signaling in renal fibroblasts

**Figure 2:**
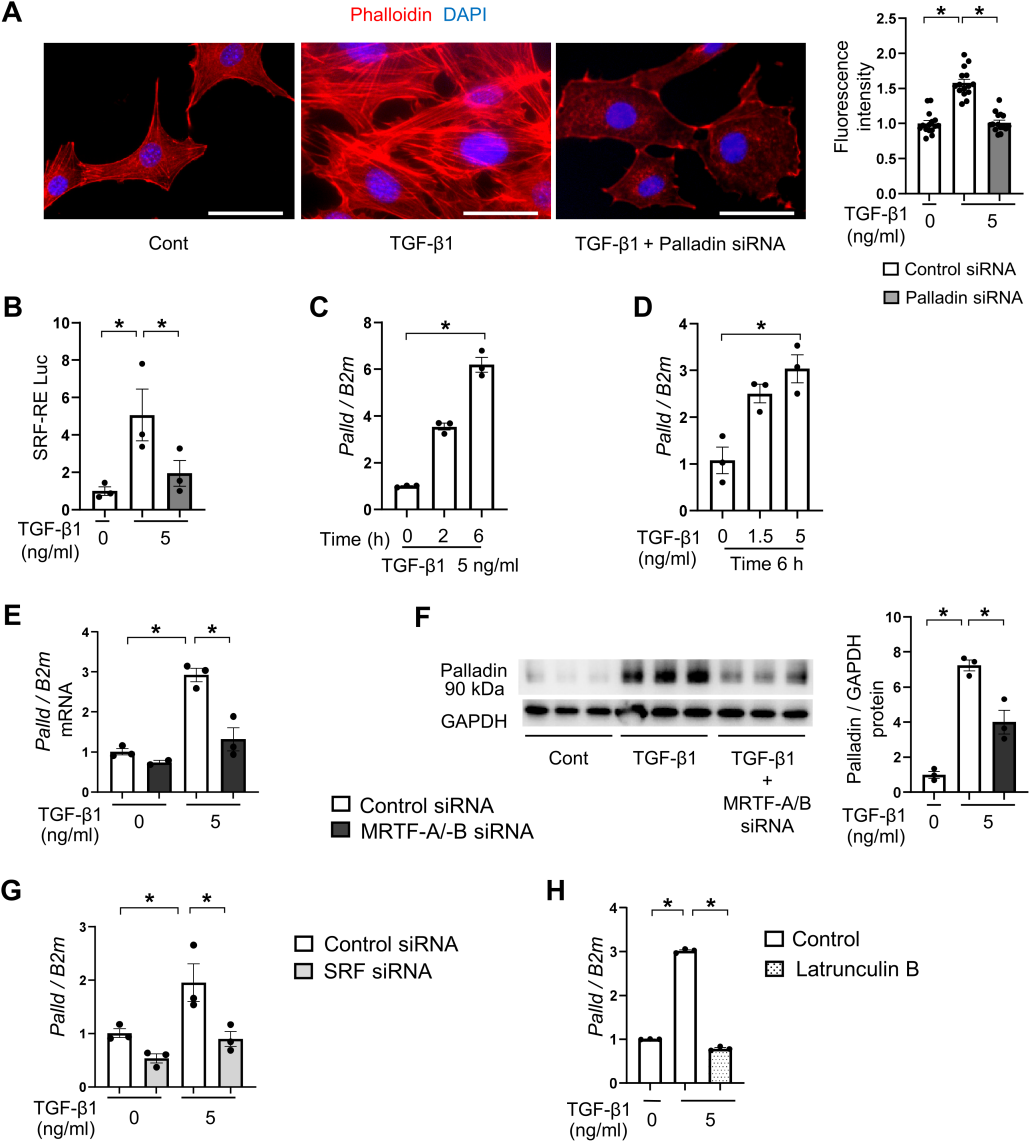
Palladin controls actin cytoskeleton and MRTF–SRF signaling in mouse renal fibroblasts, and its expression is amplified by MRTF–SRF signaling in a positive feedback loop. (A) Immunocytochemical staining for phalloidin in renal fibroblasts. Representative images of control, TGF‐β1‐stimulated renal fibroblasts, and TGF‐β1‐stimulated renal fibroblasts transfected with siRNA targeting palladin. Scale bars, 50 μm. For quantitative analysis of fluorescence intensity, five cells were randomly selected in each of three independent fields, and their mean fluorescence was measured using ImageJ software. (B) MRTF–SRF transcriptional activity in renal fibroblasts induced by TGF‐β1 stimulation was suppressed by the knockdown of palladin (*n* = 3 cell preparations/group). (C and D) Upregulation of palladin (*Palld*) mRNA in a time‐dependent (C) and dose‐dependent (D) manner induced by TGF‐β1 stimulation (*n* = 3 cell preparations/group). (E) The effect of MRTF‐A/B knockdown on palladin mRNA expression (*n* = 2 or 3 cell preparations/group). (F) Effect of MRTF‐A/B knockdown on palladin protein expression (*n* = 3 cell preparations/group). (G) Effect of SRF knockdown on palladin mRNA expression (*n* = 3 cell preparations/group). (H) Effect of pretreated latrunculin B (LB, 1 μg/ml for 30 min) on palladin mRNA expression (*n* = 3 cell preparations/group). ΔΔCT method was used to calculate relative expression of target genes, with β_2_MG (*B2m*) being the internal control. Mean ± SEM. *Statistically significant.

We next investigated the role of palladin in actin cytoskeleton signaling responses induced by TGF‐β1. Previous studies showed that palladin knockdown decreased the F‐actin/G‐actin ratio [49, 50]. Consistent with these findings, our *in vitro* loss of function studies with short interfering RNA (siRNA)‐mediated palladin knockdown demonstrated that palladin controlled actin polymerization induced by TGF‐β1 in renal fibroblasts, as assessed by phalloidin staining, compared to control siRNA (Figure 2A and supplementary material, Figure S4A). Given that MRTF–SRF signaling is activated by actin polymerization [25, 26], we then hypothesized that palladin may control MRTF–SRF signaling via actin cytoskeleton reorganization. We used a luciferase assay to assess the role of palladin in MRTF–SRF transcriptional activities in renal fibroblasts. This assay measures luciferase activity under the control of a CArG box sequence, which is the binding site of SRF, the co‐transcriptional factor of MRTF [26]. As shown in Figure 2B, knockdown of palladin suppressed SRF‐RE Luc, revealing that MRTF–SRF transcriptional activity was attenuated. Taken together, palladin drives actin polymerization and pro‐fibrotic MRTF–SRF signaling in fibroblasts.

### Palladin expression is amplified by MRTF–SRF signaling in a positive feedback loop

According to the Universal Protein Database, there are nine distinct isoforms of palladin identified so far. Of these, isoform 4, which is a 90‐kDa protein, is expressed ubiquitously in both embryonic and adult mouse tissues, especially in the stromal compartments [44]. To better understand how palladin expression is regulated in fibroblasts, we first investigated the time and dose dependency of palladin induced by TGF‐β1. As shown in Figure 2C,D, palladin was upregulated by TGF‐β1 in a time‐dependent and dose‐dependent manner at the mRNA level. Since MRTF–SRF signaling has been known to regulate the expressions of various actin‐associated proteins [22, 23, 24], we therefore hypothesized that palladin expression could be regulated by MRTF–SRF signaling in a positive feedback loop. To investigate this hypothesis, we transfected renal fibroblasts with siRNAs targeting MRTF‐A and MRTF‐B. After validating the siRNA‐induced knockdown of MRTF‐A and MRTF‐B at mRNA and protein levels (supplementary material, Figure S4B,C), we found that concomitant knockdowns of MRTF‐A and MRTF‐B reduced palladin mRNA and protein expression (Figure 2E,F). Similarly, SRF knockdown by siRNA reduced palladin expression (Figure 2G and supplementary material, Figure S4D), confirming the role of MRTF‐A/‐B/SRF in promoting palladin expression. In addition, pretreatment with CCG‐1423, an inhibitor of MRTF–SRF signaling, also reduced palladin expression induced by TGF‐β1 (supplementary material, Figure S5A,B). Furthermore, pretreatment with latrunculin B, an inhibitor of actin polymerization, significantly suppressed palladin expression in response to TGF‐β1 (Figure 2H), further validating that palladin expression was regulated by actin polymerization‐dependent MRTF–SRF signaling.

### Palladin drives myofibroblast differentiation and ECM deposition

**Figure 3:**
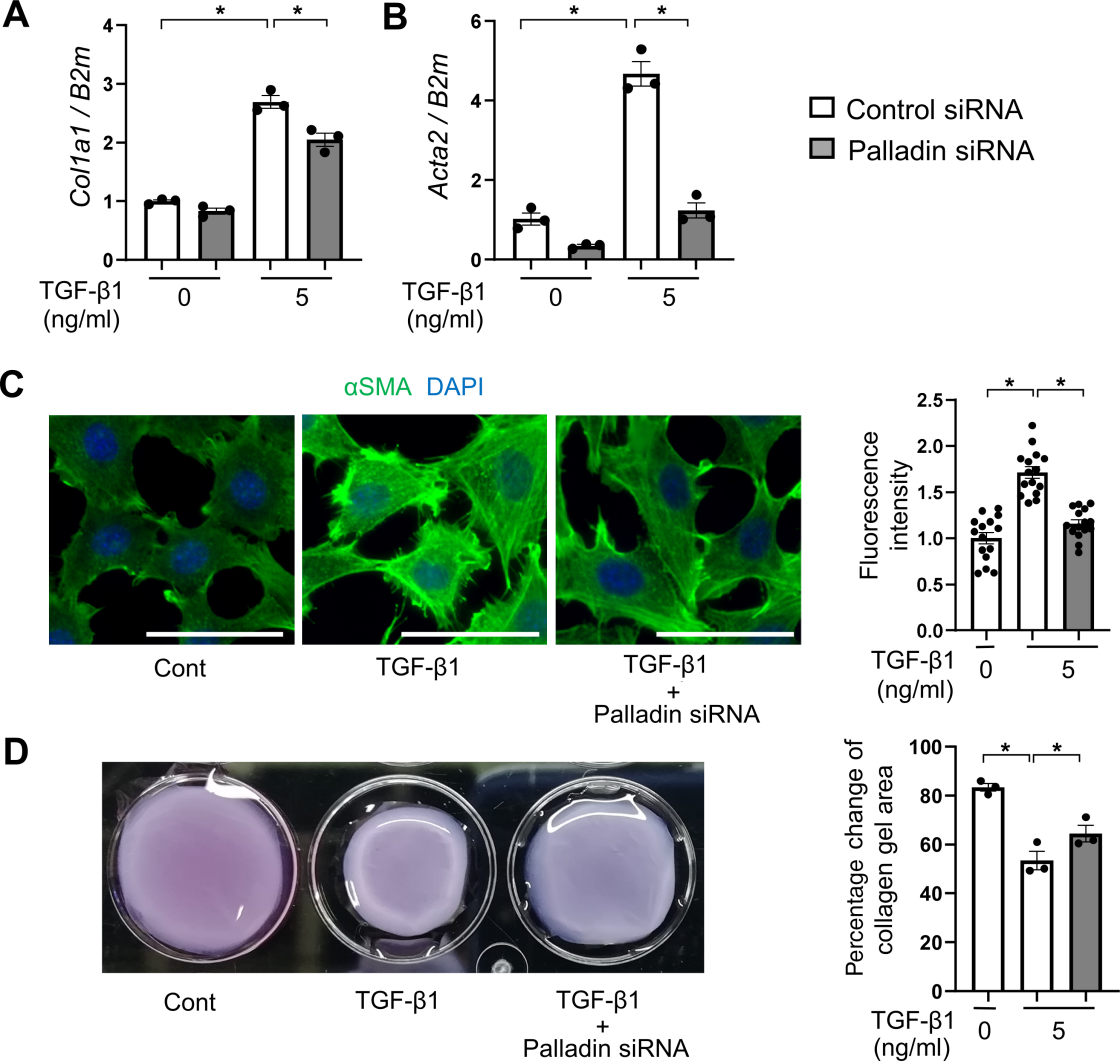
Palladin drives myofibroblast differentiation and ECM deposition. (A) Effect of palladin knockdown on *Col1a1* mRNA expression (*n* = 3 cell preparations/group). (B) Effect of palladin knockdown on *Acta2* mRNA expression (*n* = 3 cell preparations/group). (C) Immunocytochemistry: αSMA upregulation stimulated by TGF‐β1 was suppressed by palladin knockdown. Scale bars, 50 μm. For quantitative analysis of fluorescence intensity, five cells were randomly selected in each of three independent fields, and their mean fluorescence was measured using ImageJ software. (D) Collagen contraction assay: TGFβ1‐induced collagen contraction was suppressed by palladin knockdown (*n* = 3 cell preparations/group). The ΔΔCT method was used to calculate the relative expression of target genes, with β_2_MG (*B2m*) being the internal control. Mean ± SEM. *Statistically significant.

We next investigated the expression of profibrotic genes downstream of palladin and MRTF–SRF signaling. As shown in supplementary material, Figure S6, TGF‐β1 upregulated expression of *Col1a1* and *Acta2* (actin alpha2) in renal fibroblasts in a time‐dependent manner. Notably, palladin knockdown suppressed *Col1a1* and *Acta2* mRNA expression induced by TGF‐β1 in mouse renal fibroblasts (Figure 3A,B), effects mirrored at the protein level as demonstrated by immunocytochemistry (Figure 3C). In functional cell contraction assays using collagen gel, our data demonstrated that TGF‐β1‐induced collagen contraction was significantly reduced by palladin knockdown (Figure 3D). Furthermore, palladin knockdown impaired renal fibroblast proliferation in the MTT assay (supplementary material, Figure S7). Taken together, these results support the idea that palladin drives myofibroblast differentiation and ECM deposition in renal fibroblasts.

### Genetic ablation of palladin in renal fibroblasts protected mice from experimental kidney dysfunction accompanied by fibrosis

**Figure 4:**
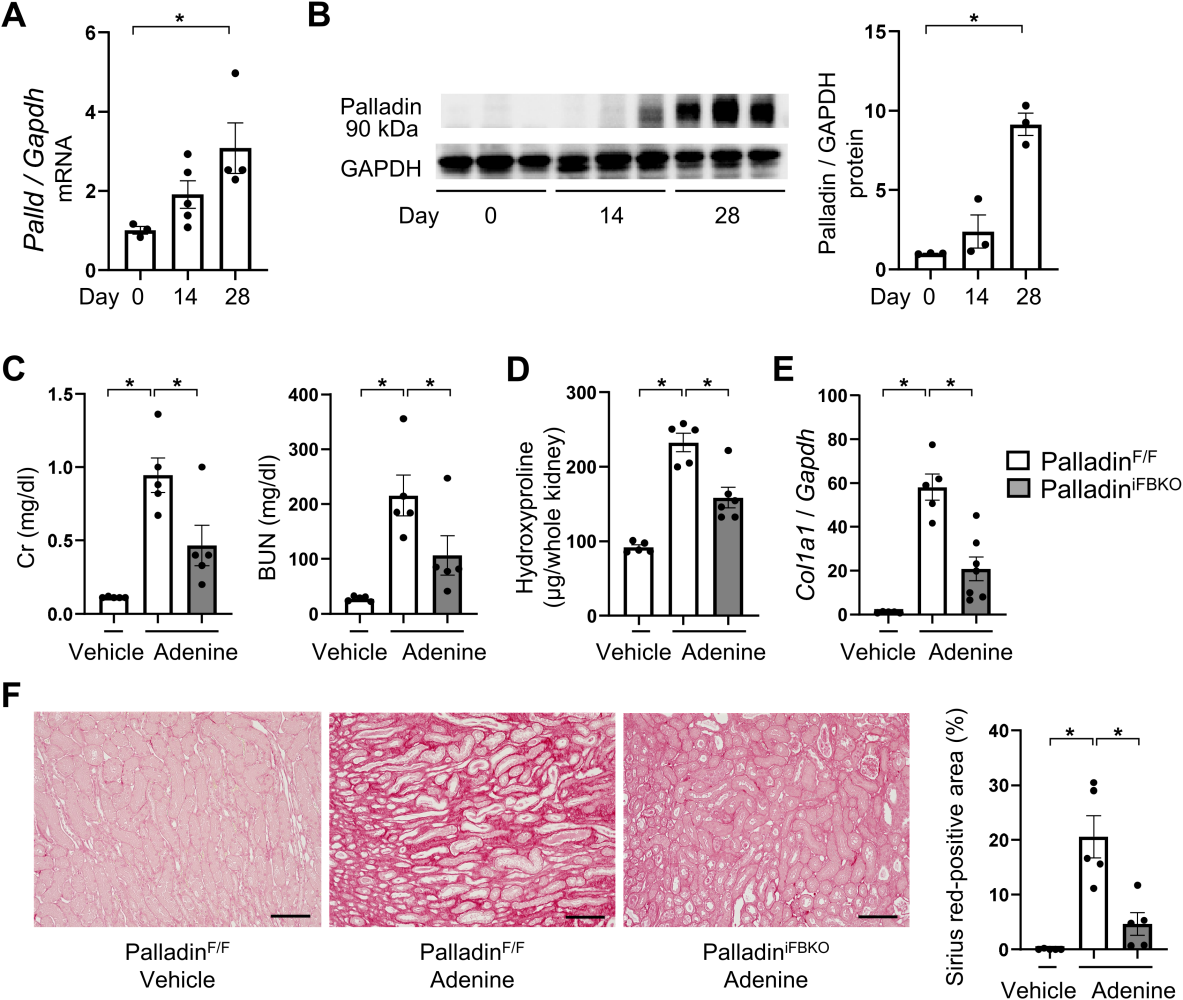
Genetic ablation of palladin in renal fibroblasts protected mice from experimental kidney dysfunction accompanied with fibrosis. (A and B) Time‐dependent upregulation of palladin (*Palld*) mRNA (A) and protein (B) in adenine‐treated palladin^F/F^ (*n* = 3–5 mice/group). (C) Serum creatinine and BUN levels in vehicle‐ or adenine‐treated palladin^F/F^ and adenine‐treated palladin^iFBKO^ (*n* = 5 mice/group). (D) Biochemical analysis of adenine‐induced kidney fibrosis: hydroxyproline content was measured in kidney following adenine administration for 28 d (*n* = 5–6 mice/group). (E) *Col1a1* expression in vehicle‐ or adenine‐treated palladin^F/F^ and adenine‐treated palladin^iFBKO^ (*n* = 5–7 mice/group). (F) Representative Sirius red‐stained kidney sections of vehicle‐ or adenine‐treated palladin^F/F^ and adenine‐treated palladin^iFBKO^, and quantitative analysis of Sirius red staining in kidney sections (*n* = 5 mice/group). Scale bars, 100 μm. The ΔΔCT method was used to calculate the relative expression of target genes, with *Gapdh* being the internal control. Mean ± SEM. *Statistically significant.

To gain insights into the role of palladin in kidney dysfunction and fibrosis *in vivo*, we adopted the well‐established murine model of kidney fibrosis based on adenine administration. Previous study showed that oral gavage of adenine induced a dose‐dependent increase in serum creatinine (Cr) and blood urea nitrogen (BUN) levels in C57BL/6 mice [34]. We performed a time‐course analysis of this model, assessing it on days 0, 14, and 28. Cr and BUN levels increased over time until day 28 (supplementary material, Figure S8A), and in addition the *Col1a1* mRNA level, hydroxyproline, and proportion of picrosirius red‐positive area increased in the same manner (supplementary material, Figure S8B–D). Our immunofluorescence staining revealed that palladin colocalized with αSMA‐expressing cells in the interstitial space of the fibrotic kidney (supplementary material, Figure S9), further suggesting a role for palladin‐dependent signaling in kidney fibrosis *in vivo*. To investigate this hypothesis, we generated novel tamoxifen‐inducible, fibroblast‐specific palladin‐deficient mice (palladin^iFBKO^) to evaluate the impact of palladin on kidney function and fibrosis *in vivo*. As shown in supplementary material, Figure S10A, palladin expression was sufficiently reduced in fibroblasts of palladin^iFBKO^ after the tamoxifen treatment. We then subjected mice without Cre‐recombinase expression (palladin^F/F^) and palladin^iFBKO^ to our kidney fibrosis model. In whole kidney samples, palladin mRNA and protein were upregulated on day 28 (Figure 4A,B), but significantly reduced in palladin^iFBKO^ (supplementary material, Figure S10B,C). Given that fibroblast‐specific deletion of palladin caused remarkable suppression of palladin in the whole kidney, we speculated that upregulation of palladin was mainly in renal fibroblasts during the development of adenine‐induced kidney fibrosis. Furthermore, immunofluorescence staining validated the notion that palladin expression in the interstitial space of the fibrotic kidney was significantly suppressed in palladin^iFBKO^ compared to palladin^F/F^ mice (supplementary material, Figure S10D). More importantly, clinically relevant markers of kidney function, including Cr and BUN levels, were remarkably decreased in palladin^iFBKO^ compared to palladin^F/F^ mice (Figure 4C). In addition, palladin^iFBKO^ mice showed marked decreases in markers of fibrosis including hydroxyproline content, COL1A1 expression, and the proportion of picrosirius red‐positive area (Figure 4D–F). Consistent with our results in the adenine‐induced nephropathy model, fibroblast‐specific palladin deficiency protected against folic acid‐induced kidney fibrosis (supplementary material, Figure S11A,B). Collectively, our data indicate that genetic ablation of palladin in renal fibroblasts protects mice from experimental kidney dysfunction accompanied by fibrosis.

### Fibroblast‐specific deficiency of palladin prevents myofibroblast formation *in vivo*

**Figure 5:**
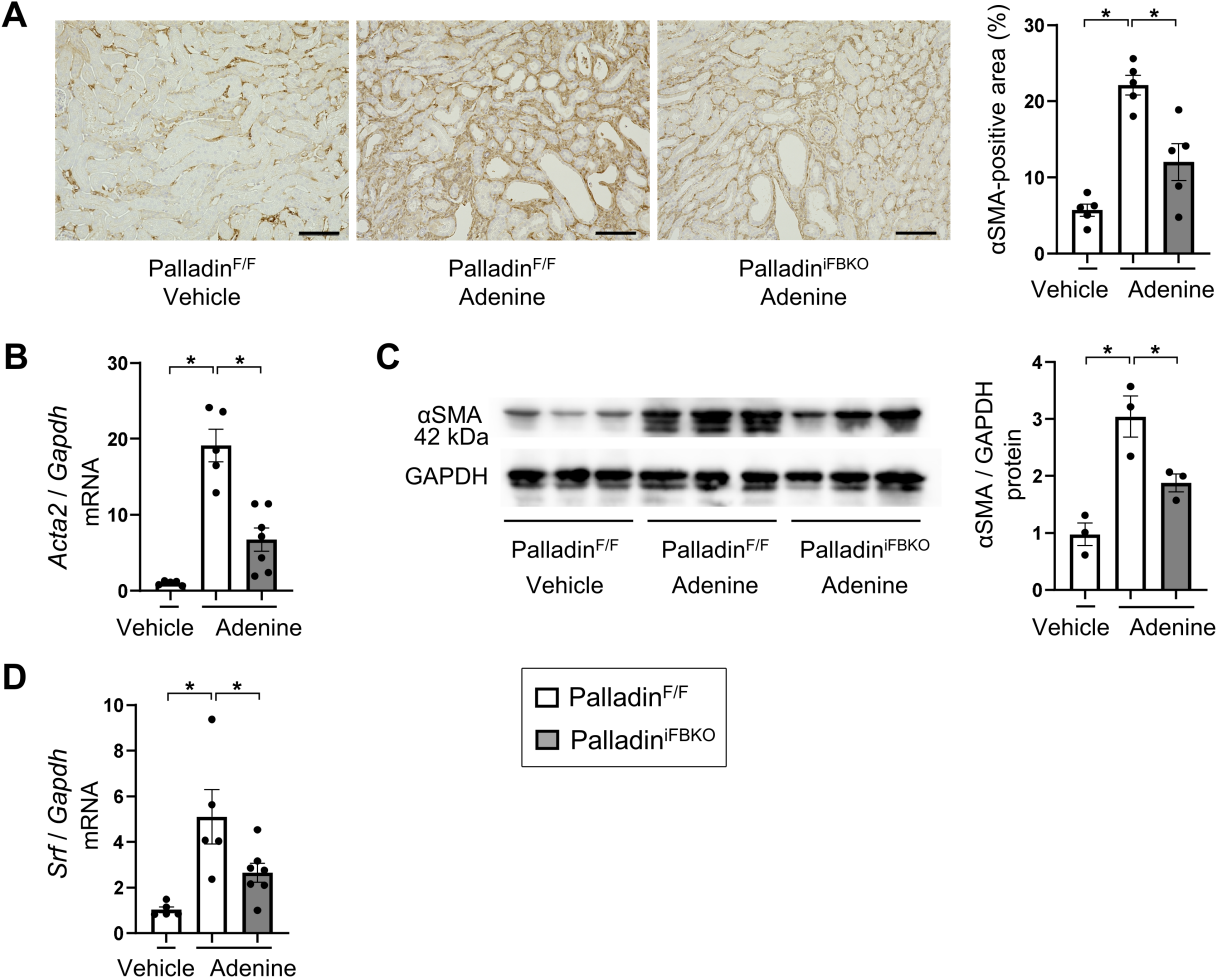
Fibroblast‐specific deficiency of palladin prevents myofibroblast formation *in vivo*. (A) Representative αSMA‐stained kidney sections of vehicle‐ or adenine‐treated palladin^F/F^ and adenine‐treated palladin^iFBKO^, and quantitative analysis of αSMA staining in kidney sections (*n* = 5 mice/group). Scale bars, 100 μm. (B and C) Expression of (B) *Acta2* mRNA and (C) protein in vehicle‐ or adenine‐treated palladin^F/F^ and adenine‐treated palladin^iFBKO^ (*n* = 5–7 mice/group). Palladin and αSMA were detected sequentially on the same membrane using a strip‐and‐reprobe protocol; GAPDH was probed last as the loading control. Consequently, the GAPDH panel is identical to that shown in supplementary material, Figure S10C. Densitometry for palladin and αSMA was normalized to this GAPDH. (D) *Srf* mRNA expression in vehicle‐ or adenine‐treated palladin^F/F^ and adenine‐treated palladin^iFBKO^ (*n* = 5–7 mice/group). The ΔΔCT method was used to calculate the relative expression of target genes, with *Gapdh* being the internal control. Mean ± SEM. *Statistically significant.

To elucidate the mechanisms by which palladin in fibroblasts regulates renal fibrosis, we investigated the expression of myofibroblast marker αSMA in fibrotic kidneys. Our data demonstrated that *Acta2* mRNA was upregulated time‐dependently in our murine experimental model (supplementary material, Figure S12A). At day 28, the peak of fibrosis markers, the number of αSMA‐positive myofibroblasts had significantly reduced in palladin^iFBKO^ compared to palladin^F/F^ mice, as demonstrated by immunohistochemistry (Figure 5A). Furthermore, *Acta2* mRNA and αSMA protein levels had similarly decreased in palladin^iFBKO^ compared to palladin^F/F^ mice (Figure 5B,C). Moreover, fibroblast‐specific palladin deficiency attenuated folic acid‐induced myofibroblast differentiation (supplementary material, Figure S11C). Taken together, these results support the notion that palladin contributed to the development of kidney fibrosis by regulating myofibroblast formation.

### Fibroblast‐specific deficiency of palladin suppressed MRTF–SRF signaling *in vivo*

To verify the effect of palladin on MRTF–SRF signaling in the course of kidney fibrosis, we investigated SRF expression, which is regulated by MRTF–SRF signaling. As shown in supplementary material, Figure S12B, SRF was upregulated in a time‐dependent manner. Palladin^iFBKO^ revealed *Srf* mRNA downregulation (Figure 5D), confirming the role of this pathway *in vivo*.

### Expression of palladin–MRTF–SRF correlates with kidney function and fibrosis in human disease

**Figure 6:**
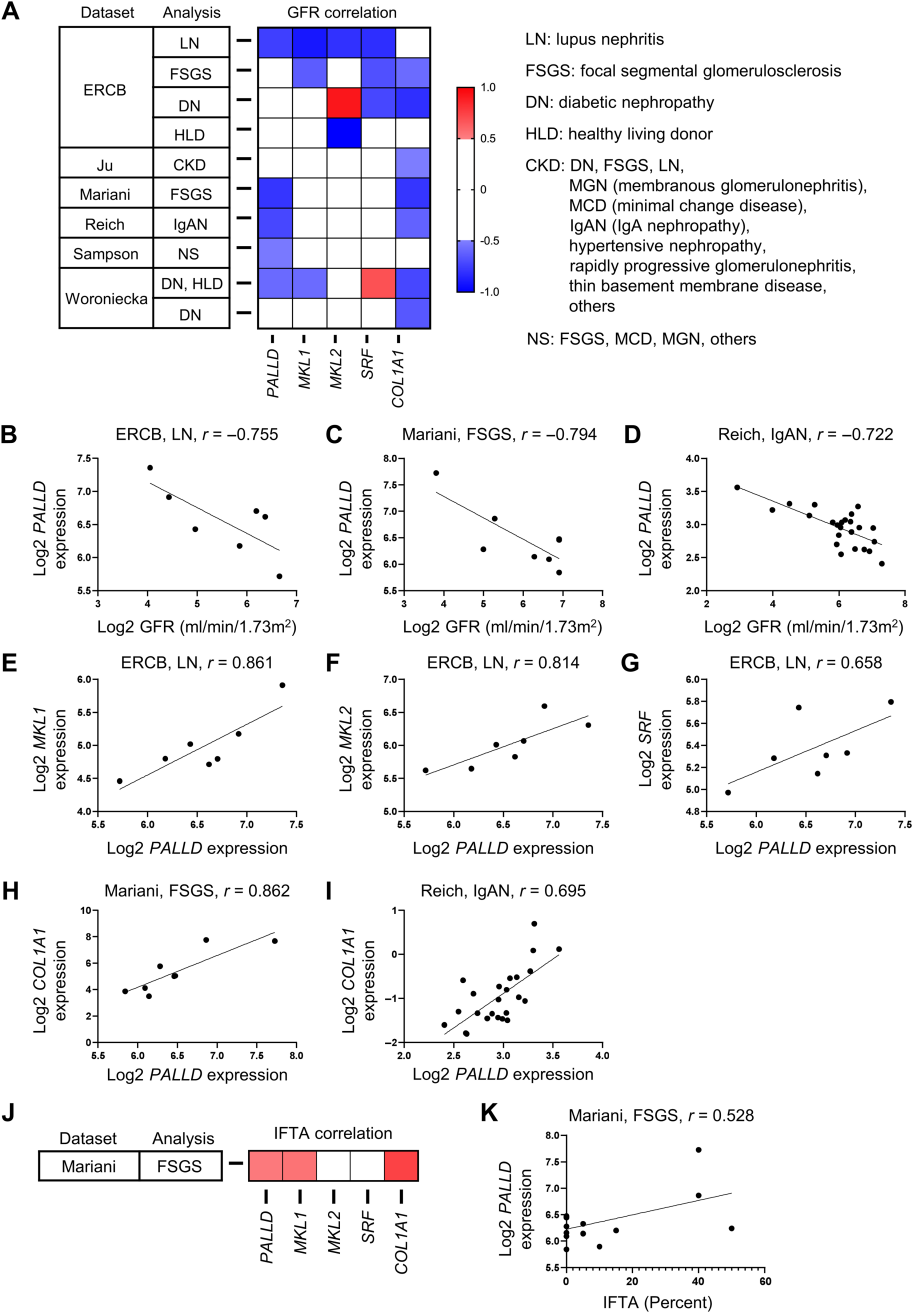
Expression of palladin–MRTF–SRF correlates with kidney function and fibrosis in human disease. (A) Summary of NephroSeq. Version 5 data filtered for tubulointerstitial tissue, significant results (*p* < 0.05), and Pearson's correlation coefficient, |*r*| > 0.5 (GFR). (B–D) Scatter plots revealing correlation between GFR and *PALLD* expression in (B) human lupus nephritis, (C) FSGS, and (D) IgA nephropathy. (E–G) Scatter plot revealing correlation of *PALLD* with (E) *MKL1*, (F), *MKL2*, and (G) *SRF* in human lupus nephritis. (H and I) Scatter plot revealing correlation between *PALLD* and *COL1A1* expression in (H) human FSGS and (I) IgA nephropathy. (J) Summary of NephroSeq. Version 5 data filtered for tubulointerstitial tissue: significant results (*p* < 0.05), and Pearson's correlation coefficient, |*r*| > 0.5 (IFTA). (K) Scatter plot revealing correlation between IFTA and *PALLD* expression in human FSGS.

To further confirm the importance of palladin in human kidney function and fibrosis, we analyzed the Nephroseq database [36], which is a publicly available database with various gene signatures in human kidney diseases. We first investigated palladin (*PALLD* gene), MRTF–SRF signaling (*MKL1*, *MKL2*, and *SRF* genes), and COL1A1 (*COL1A1* gene), in a GFR analysis. The extraction condition included tubulointerstitium, *p* < 0.05, and Pearson's correlation coefficient for |*r*| > 0.5. Consistent with our kidney biopsy studies in humans, *PALLD* was negatively correlated with GFR across a wide spectrum of diseases such as lupus nephritis, focal segmental glomerulonephritis (FSGS), and IgA nephropathy (Figure 6A–D). In lupus nephritis, *MKL1*, *MKL2*, and *SRF* also negatively correlated with GFR, and *PALLD* was shown to negatively correlate with GFR. Interestingly, *PALLD* was strongly correlated with *MKL1*, *MKL2*, and *SRF* in human lupus nephritis samples (Figure 6E–G). *PALLD* was also correlated with *COL1A1* in human FSGS and IgA nephropathy samples (Figure 6H,I). Under the same extraction conditions, we also evaluated *PALLD*, *MKL1*, *MKL2*, *SRF*, and *COL1A1* in kidney fibrosis, downloading for interstitial fibrosis and tubular atrophy (IFTA) analysis. *PALLD*, along with *MKL1* and *COL1A1*, showed to be positively correlated with the proportion of IFTA in human FSGS samples (Figure 6J,K). Collectively, these results supported the role of palladin–MRTF–SRF axis in the development of human kidney diseases and fibrosis.

## Discussion

Here, we used cultured cells and mouse and human kidney samples to demonstrate that palladin, an actin‐associated protein, was upregulated in kidney fibrosis. Proteome analysis revealed actin‐associated proteins were related to GFR and COL1A1 expression. Of these, *Palld* is a TGF‐β1‐dependent target gene highly expressed in the kidney interstitial space of patients with low eGFR. In renal fibroblasts treated with TGF‐β1, we found that palladin knockdown suppressed actin polymerization and MRTF–SRF activity. Our studies also revealed that palladin expression was controlled by actin polymerization‐dependent MRTF–SRF signaling in a positive feedback loop. Genetic blockade of the palladin–MRTF–SRF axis reduced myofibroblast accumulation and ECM deposition *in vitro*. *In vivo*, genetic ablation of palladin in fibroblasts protected mice from kidney dysfunction and fibrosis, while reducing myofibroblast differentiation and SRF expression. Lastly, our analyses of human samples, using a publicly available database, showed that the palladin–MRTF–SRF pathway was strongly associated with kidney function in various human kidney diseases. Taken altogether, palladin builds profibrotic circuits of the palladin–MRTF–SRF axis through actin cytoskeleton reorganization. These perpetuating networks may contribute to renal impairment coupled with fibrosis by inducing both COL1A1 and αSMA expression, resulting in ECM deposition and myofibroblast differentiation (supplementary material, Figure S13).

Actin rearrangement is a crucial process in fibroblast activation. Our results demonstrate that palladin, an actin cross‐linking protein, is a key player in kidney dysfunction and fibrosis induced by TGF‐β1. It has been indicated that palladin works as an actin scaffolding protein by binding to direct and indirect regulators of actin filaments [18, 51, 52, 53, 54, 55, 56]. We demonstrated that palladin knockdown diminished actin polymerization induced by TGF‐β1. Therefore, actin rearrangement in response to TGF‐β1 is significantly influenced by palladin.

Actin cytoskeleton and its related signals are associated with numerous indications. Thus far, we have shown that the actin cytoskeleton–MRTF–SRF signaling is a driver of fibrosis [22, 23, 24]. Here, our proteomics approach demonstrated the importance of actin‐associated proteins in kidney fibrosis. Of these, palladin, a TGF‐β1‐dependent actin‐associated protein, created the profibrotic circuit with MRTF–SRF signaling, which perpetuated ECM deposition and myofibroblast differentiation. We have already shown that this persistent fibrotic process causes ECM stiffness following additional ECM production and stabilization, creating the amplification loop of stiffer ECM [24, 57]. It has been reported that MRTF is potentially an important transcription factor in the mechanotransduction pathway [8, 57]. Our data support the idea that palladin may regulate MRTF–SRF signaling, aggravating the profibrotic mechanical feedback loop through actin cytoskeletal remodeling. Further research is required to clarify the precise mechanical mechanisms by which palladin controls mechanotransduction driven by ECM stiffness.

In patients, we showed that palladin was upregulated in the kidney interstitium of CKD patients with low eGFR. Regarding the involvement of palladin in the glomerulus, ablation of palladin in podocytes developed a higher level of proteinuria with mild foot process effacement after nephrotoxic serum injection [58]. In addition, cardiomyocyte‐specific palladin deletion in mice results in dilated cardiomyopathy with intercalated disc abnormalities and fibrosis [59]. Given the different roles of palladin in each cell and organ, palladin may function as a biomarker in humans. The Nephroseq database revealed that palladin expression in tubulointerstitium was negatively correlated with GFR across a wide range of kidney diseases. However, it remains unclear whether interstitial palladin expression is correlated with clinicopathological findings. Therefore, further study is needed to explore the possibility of palladin as a biomarker in the kidney.

In summary, we have provided new insights into the involvement of palladin in kidney dysfunction and fibrosis through positive feedback loops in the palladin–MRTF–SRF axis. In addition, fibroblast‐specific palladin deletion remarkably attenuated kidney dysfunction and fibrosis. Therefore, the palladin suppression therapy targeting renal fibroblast sheds light on novel therapeutic strategies for CKD.

## Author contributions statement

NS, DL, TW and YI conceived and designed the study. NY, NS and YI curated the data, and NY and NS performed the formal analyses. NY, NS, YY, JT, SH, TD, MK, HA and SO developed the methodology. NY, NS, YY, DK, DH, TM, AK, KS, KH, TY, AT, TM, MO, SN, SK, AH, MS, JT, SH, TD, AM, HI, MK, HA and SO performed the experiments. NS and YI administered the project and provided resources. NS, YY, TW and YI supervised the work. NS, YI and SO acquired funding. NY, NS, TD, SO and DL wrote the original draft, and NS, DL, TW and YI reviewed and edited the manuscript. NY, NS and YI validated the results. All authors reviewed and approved the final version of the manuscript.

## Supporting information


**Supplementary materials** **and methods**
**Figure S1.** Genotyping information and protocol of kidney fibrosis model based on adenine. administration
**Figure S2**. Overview of proteome analysis
**Figure S3.** Palladin is intracellular and colocalizes with αSMA‐expressing cells in interstitium of human kidney
**Figure S4.** Validation of siRNA transfection efficiency in renal fibroblasts
**Figure S5.** Palladin expression is suppressed by CCG at mRNA and protein levels
**Figure S6.** TGF‐β1 enhances expression of *Col1a1* and *Acta2* mRNA in mouse renal fibroblasts
**Figure S7.** Palladin regulates fibroblast proliferation
**Figure S8.** Adenine administration induces kidney dysfunction and fibrosis in mice
**Figure S9.** Palladin colocalizes with αSMA‐expressing cells in the interstitial space of the fibrotic kidney
**Figure S10.** Palladin is remarkably suppressed in the whole kidney as well as fibroblasts of palladin^iFBKO^
**Figure S11.** Fibroblast‐specific palladin deletion ameliorates kidney fibrosis in folic acid‐induced nephropathy
**Figure S12.** Adenine administration upregulates expressions of *Acta2* and *Srf*
**Figure S13.** Proposed schema for profibrotic circuits of palladin–MRTF–SRF axis in pathogenesis of kidney fibrosis
**Table S1.** Primers for reverse transcription‐quantitative polymerase chain reaction (RT‐qPCR)
**Table S2.** List of primary antibodies used for immunohistochemistry, immunocytochemical analyses assay, and western blotting

## Data Availability

The raw data have been deposited to the ProteomeXchange Consortium via the JPOST partner repository with the dataset identifier JPST003595 (PXD060453; https://proteomecentral.proteomexchange.org/cgi/GetDataset?ID=PXD060453). Publicly available data sets were obtained from https://www.nephroseq.org/resource/login.html.
