# Supplementary material for: Fibroblast‐specific palladin drives kidney fibrosis via MRTF–SRF signaling
Source: J Pathol. 2025 Oct 24;268(1):40–53. doi: 10.1002/path.6485 (PMC12699245; doi:10.1002/path.6485)
Supplement: Supplementary file 1 — Supplementary materials and methods Figure S1. Genotyping information and protocol of kidney fibrosis model based on adenine. administration Figure S2. Overview of proteome analysis Figure S3. Palladin is intracellular and colocalizes with αSMA‐expressing cells in interstitium of human kidney Figure S4. Validation of siRNA transfection efficiency in renal fibroblasts Figure S5. Palladin expression is suppressed by CCG at mRNA and protein levels Figure S6. TGF‐β1 enhances expression of Col1a1 and Acta2 mRNA in mouse renal fibroblasts Figure S7. Palladin regulates fibroblast proliferation Figure S8. Adenine administration induces kidney dysfunction and fibrosis in mice Figure S9. Palladin colocalizes with αSMA‐expressing cells in the interstitial space of the fibrotic kidney Figure S10. Palladin is remarkably suppressed in the whole kidney as well as fibroblasts of palladiniFBKO Figure S11. Fibroblast‐specific palladin deletion ameliorates kidney fibrosis in folic acid‐induced nephropathy Figure S12. Adenine administration upregulates expressions of Acta2 and Srf Figure S13. Proposed schema for profibrotic circuits of palladin–MRTF–SRF axis in pathogenesis of kidney fibrosis Table S1. Primers for reverse transcription‐quantitative polymerase chain reaction (RT‐qPCR) Table S2. List of primary antibodies used for immunohistochemistry, immunocytochemical analyses assay, and western blotting [file PATH-268-40-s001.docx]

**Fibroblast-specific palladin drives kidney fibrosis via MRTF–SRF signaling**

N Yamamoto *et al. J Pathol* <https://doi.org/10.1002/path.6485>

**Supplementary materials and methods**

**Supplementary Figures S1–S13**

**Supplementary Tables S1 and S2**

Reference numbers refer to the main text list

**Quantitative PCR analysis**

Total cellular RNA was isolated from primary cells and renal tissue using ISOSPIN cell and tissue RNA (NIPPON Gene, Tokyo, Japan) according to the manufacturer's protocol. Complementary DNA (cDNA) was synthesized using a high-capacity RNA-to-cDNA kit (Applied Biosystems, Foster City, CA, USA). SYBR Green PCR Master Mix (Bio-Rad, Hercules, CA, USA) was used for quantitative real-time PCR. Quantitative real-time PCR analysis using a ViiA™ 7 Real-Time PCR System (Thermo Fisher Scientific, Waltham, MA, USA) was performed. The ΔΔCT method was used to calculate the relative gene expression of target genes relative to the internal controls. All the primers we used in this study are listed in supplementary material, Table S1. For the adenine nephropathy experiments, five palladin^F/F^ mice were allocated to vehicle or adenine treatment groups. Seven palladin^iFBKO^ mice were assigned to the adenine group.

**Transfection with siRNAs**

ON-TARGET plus SMART pool siRNAs targeting mouse palladin (Horizon Discovery, Cambridge, UK), MRTF-A, MRTF-B, and SRF (Horizon Discovery) were used. We used ON-TARGET plus nontargeting pool siRNAs (Horizon Discovery) as a nonspecific control. Cells were transfected using lipofectamine RNAiMAX Reagant (Thermo Fisher Scientific) following the manufacturer’s protocol.

**Western blot analysis**

Samples were solubilized in 1×RIPA Lysis Buffer (Merck Millipore, Billerica, MA, USA) supplemented with phosphatase inhibitor and protease inhibitor (Roche, Mannheim, Germany). Cellular and tissue lysates were separated by SDS-polyacrylamide gel electrophoresis (Wako, Osaka, Japan) and transferred to polyvinylidene difluoride membranes (Merck Millipore), followed by immunoblotting. Protein expression was evaluated using an anti-palladin antibody (Proteintech, Rosemont, IL, USA) at 1:3,000 dilution and anti-αSMA antibody (Abcam, Cambridge, MA, USA) at 1:20,000 dilution for 1 h at room temperature, followed by secondary antibody at 1:3,000 dilution for 30 min at room temperature (Cell Signaling Technology, Danvers, MA, USA). The analysis of blotted membranes was conducted using ChemiDoc Imaging System (Bio-Rad) and ImageJ software (National Institutes of Health, Rockville, MD, USA). Detailed information on antibodies is provided in Table S2. For the mouse experiments, three biological samples per treatment group were randomly selected and analyzed.

**Immunocytochemical analyses**

Actin polymerization and αSMA expression were assessed by immunocytochemistry as previously described [52]. In brief, cells were fixed with 4% PFA for 10 min at 4 ℃. Blocking was performed with 0.1% Triton X-100 and 10% normal goat serum (Millipore, Burlington, MA, USA) in PBS for 1 h at room temperature. Primary antibodies were diluted in blocking solution and incubated overnight at 4 ℃. The cells were incubated with secondary antibodies for 1 h at room temperature. Rabbit anti-α-smooth muscle actin (αSMA) (Abcam) at 1:400 dilution and Actin-Stain 555 (Cytoskeleton, Denver, CO, USA) at a 1:140 dilution were used for primary staining. The secondary antibodies were DyLight 488 (Abcam) conjugated. All the antibodies we used in this study are listed in Table S2. For quantitative analysis of fluorescence intensity, five cells were randomly selected in each of three independent fields, and their mean fluorescence was measured using ImageJ software.

**Luciferase reporter assay**

Luciferase reporter assays were performed to examine the activity of MRTF/SRF-dependent gene transcription using pGL4.34 and pGL4.74 (Promega, Madison, WI, USA), as previously described [60]. Luciferase levels were measured using Dual-Glo luciferase assay system (Promega).

**Cell contraction assay**

Collagen gel contraction assay was performed following the manufacturer's protocol (Cell Biolabs, San Diego, CA, USA). In brief, the suspension of renal fibroblasts was mixed with collagen solution at a ratio of 1:4. After starvation for 16 h, collagen gels were gently released from the culture dishes and stimulated with 5 ng/ml TGF-β1 (R&D Systems, Minneapolis, MN, USA) for 24 h. The change of collagen gel size was analyzed using ImageJ software.

**Cell proliferation assay**

Cell Counting Kit-8 was used to assess cell proliferation according to the manufacturer’s protocol (Dojindo Laboratories, Kumamoto, Japan). In brief, renal fibroblasts were suspended and seeded into a 96-well plate. After starvation for 16 h, RFB were stimulated with 5 ng/ml TGF-β1 for 22 h and incubated with CCK-8 reagent for 2 h at 37 °C. The amount of color developed was measured at 450 nm using a microplate reader.

**Isolation of primary dermal fibroblasts from mice**

Dermal fibroblasts were obtained as previously described [61]. In brief, skin samples of 1 cm^3^ were harvested from mice that were clean and shaved. The tissue was cut into 1 mm^3^ pieces, placed in sterile plastic dished, and cultured in DMEM with 10% FBS, 1% sodium pyruvate, 1% nonessential amino acid mixture, 1% penicillin/streptomycin stock solution, and 1% L-glutamine at 37 ℃ in a 5% CO_2_ humidified atmosphere. After 2 weeks of incubation, total cellular RNA was extracted from the outgrowth of dermal fibroblasts, and the expression of palladin (*Palld*) was analyzed.

**Kidney histopathology**

One portion of renal tissue from each mouse was fixed in 10% buffered formalin (pH 7.2), embedded in paraffin, cut into 1.5-μm sections, and Sirius red, αSMA, and palladin staining performed. Picrosirius red staining of the kidney sections was performed following the supplier’s instructions (Polysciences, Warrington, PA, USA). Myofibroblasts were identified in tissue samples using anti-αSMA antibody (Abcam) at a 1:2,000 dilution overnight at 4 °C, followed by Histofine Simple Stain Mouse MAX-PO (Rabbit) kit for 30 min (Nichirei Bioscience, Tokyo, Japan). Palladin expression was evaluated using an anti-palladin antibody (Proteintech, Rosemont, IL, USA) at a 1:200 dilution for 1 h at room temperature. Antibody-stained cells were visualized using Dylight 488-conjugated streptavidin secondary antibody (Vector Laboratories, Newark, CA, USA). To determine the colocalization of palladin and αSMA, kidney sections from palladin^F/F^ were costained for palladin and αSMA using a M.O.M. kit (Vector Laboratories). Sections were incubated with anti-αSMA antibody (Santa Cruz Biotechnology, Dallas, TX, USA) at a 1:200 dilution for 40 min at room temperature and anti-palladin antibody (Proteintech) at a 1:200 dilution for 1 h at room temperature. Mean positive areas were determined within the area of cortex and outer medulla in the kidney section using ImageJ software. The antibodies are listed in supplementary material, Table S2. For the adenine nephropathy experiments, five biological samples per treatment group were randomly selected and analyzed.

**Kidney function assay**

Blood samples were collected from the inferior vena cava of mice. The blood was centrifuged at 3,000 rpm for 5 min to separate serum. Blood urea nitrogen (BUN) and serum creatinine levels were measured using an automated analyzer (JCA-BM6050; Japan Electron Optics Laboratory, Tokyo, Japan). For the adenine nephropathy experiments, five palladin^F/F^ mice were allocated to vehicle or adenine treatment groups. Six palladin^iFBKO^ mice were assigned to the adenine group; however, one palladin^iFBKO^ mouse could not be sampled due to unsuccessful blood collection.

**Hydroxyproline assay**

Whole left kidneys were homogenized in 500 µl PBS and hydrolyzed in 6 M HCl at 120 °C for 16 h. Aliquots of 25 µl were added to 1 ml of 1.4% chloramine T (Sigma-Aldrich), 10% *n*-propanol, and 0.25 M sodium acetate. After 20 min of incubation at room temperature, 1 ml Erlich's solution (1 M *p*-dimethylaminobenzaldehyde in 70% *n*-propanol, 20% perchloric acid) was added, and a 15-min incubation at 65 °C was performed. Absorbance was measured at 550 nm, and the amount of hydroxyproline was determined against a standard curve. For the adenine nephropathy experiments, five palladin^F/F^ mice were allocated to vehicle or adenine treatment groups. Six palladin^iFBKO^ mice were assigned to the adenine group.

**Supplementary Figures S1–S13**

**
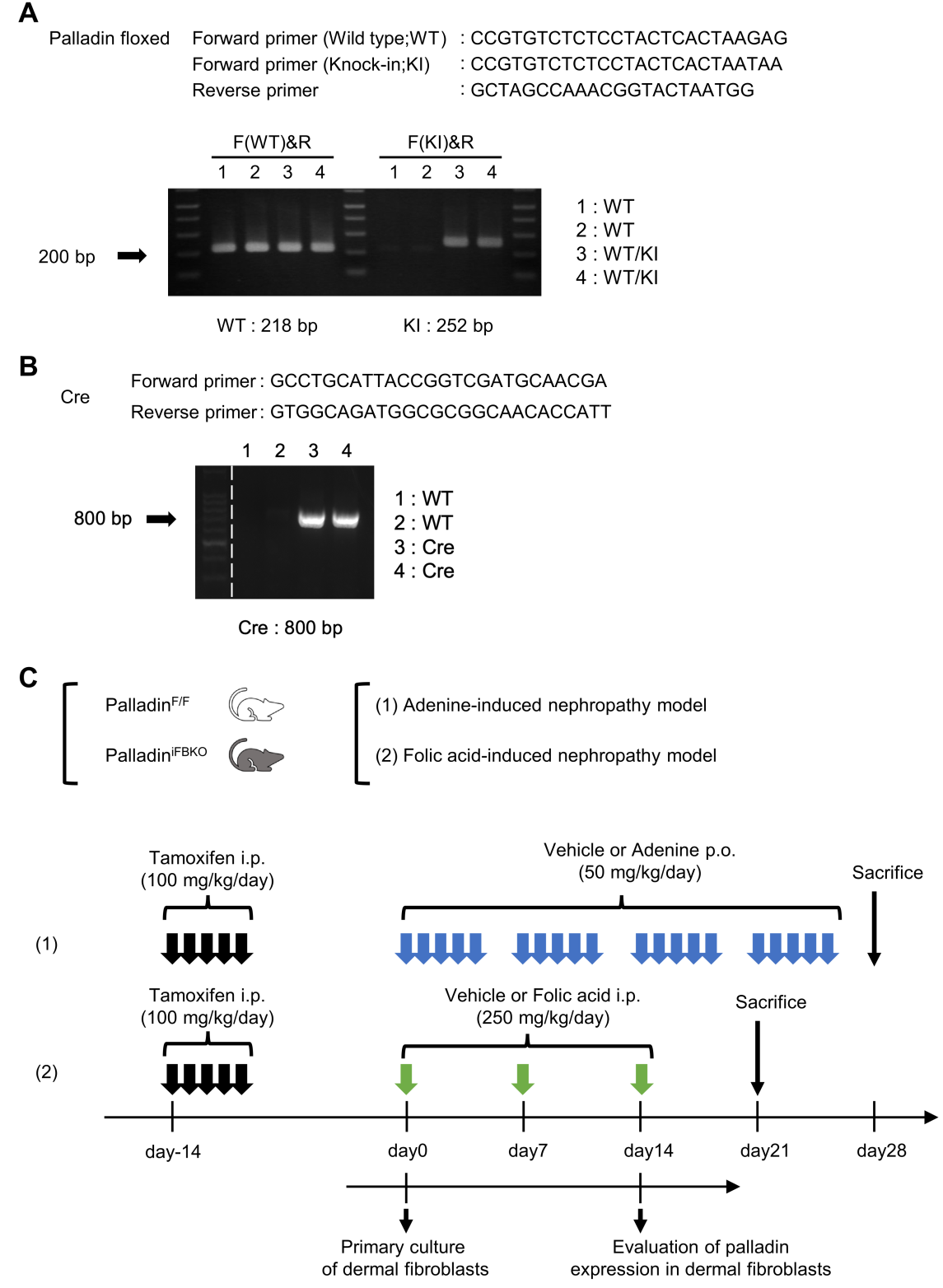
**

**Figure S1. Genotyping information and protocol of kidney fibrosis model based on adenine administration.** (A) Primer sequences for palladin floxed mice with the genotyping image. (B) Primer sequences for Col1a2-Cre-ER(T) mice with genotyping image. The molecular weight marker lane was repositioned from the same gel image to the left edge for clarity. (C) Schematic diagram for kidney fibrosis model and evaluating efficiency of palladin deletion in dermal fibroblasts.

**
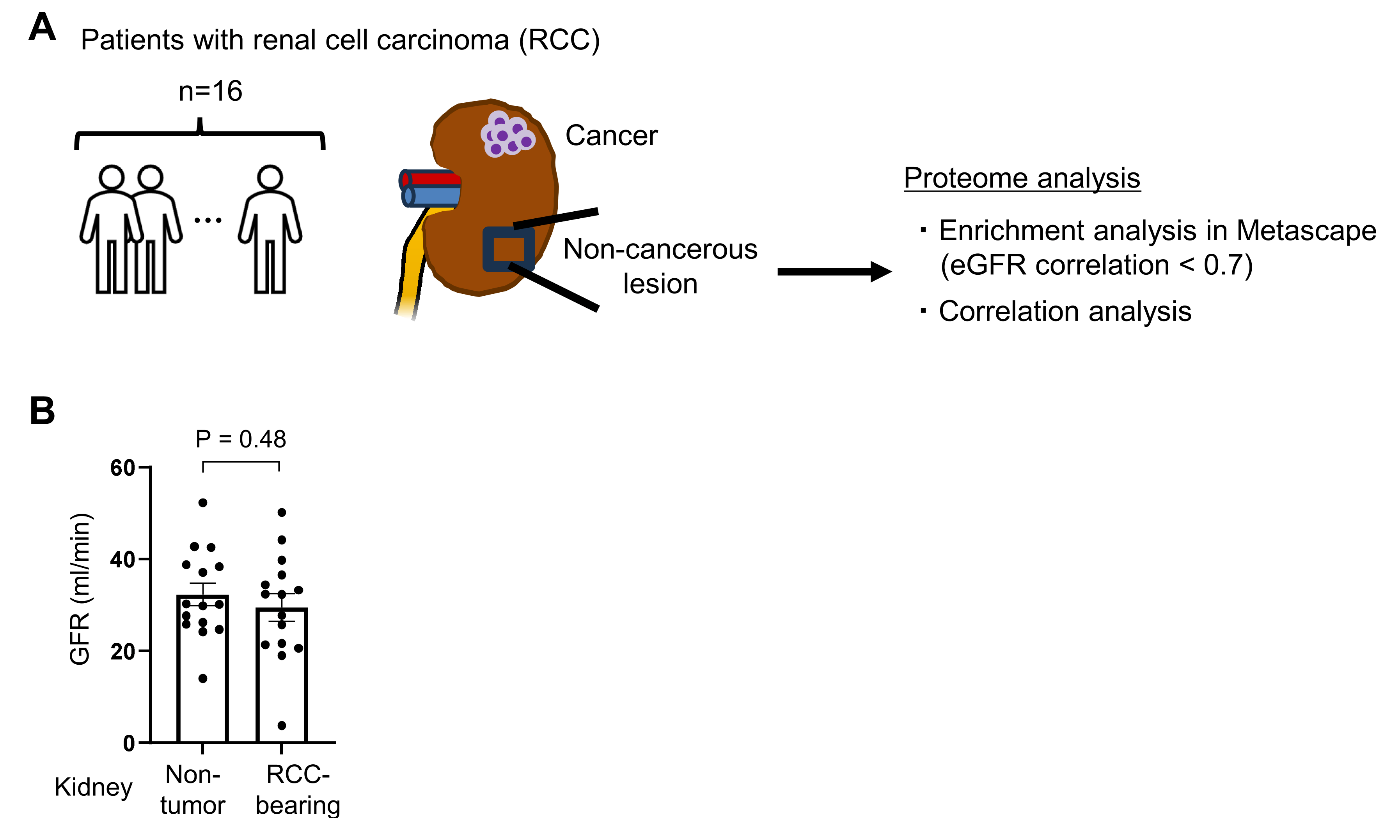
**

**Figure S2. Overview of proteome analysis.** (A) Workflow for collection of noncancerous kidney tissue from patients with renal cell carcinoma (RCC). (B) Comparison of GFR between RCC-bearing kidney and contralateral nontumor kidney.


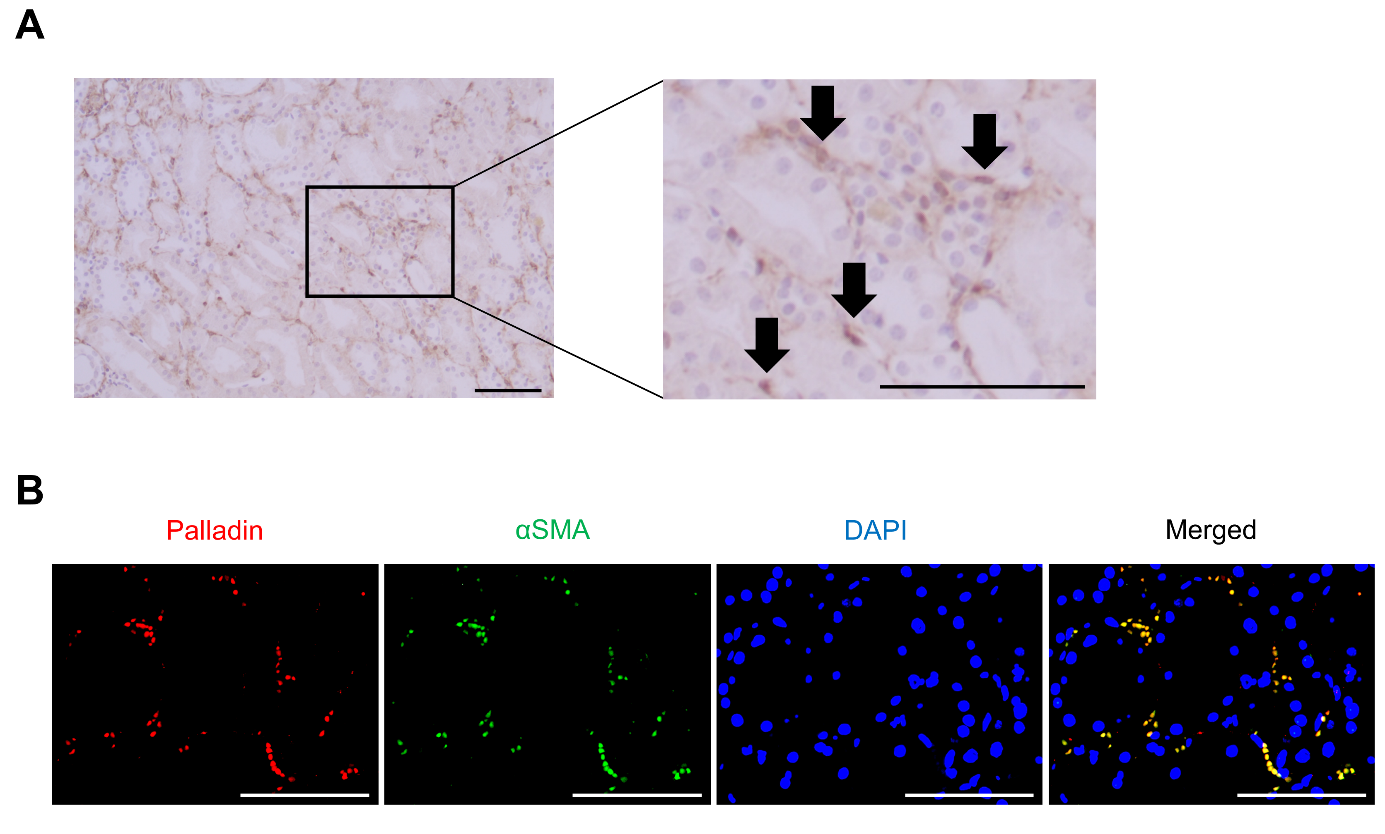


**Figure S3. Palladin is intracellular and colocalizes with αSMA-expressing cells in interstitium of the human kidney.** (A) Representative images of human kidney biopsy sections stained for palladin using 3,3’-diaminobenzidine (DAB). Scale bars, 100 µm. (B) Representative images of human kidney sections costained with anti-palladin and anti-αSMA antibodies. Scale bars, 100 µm.


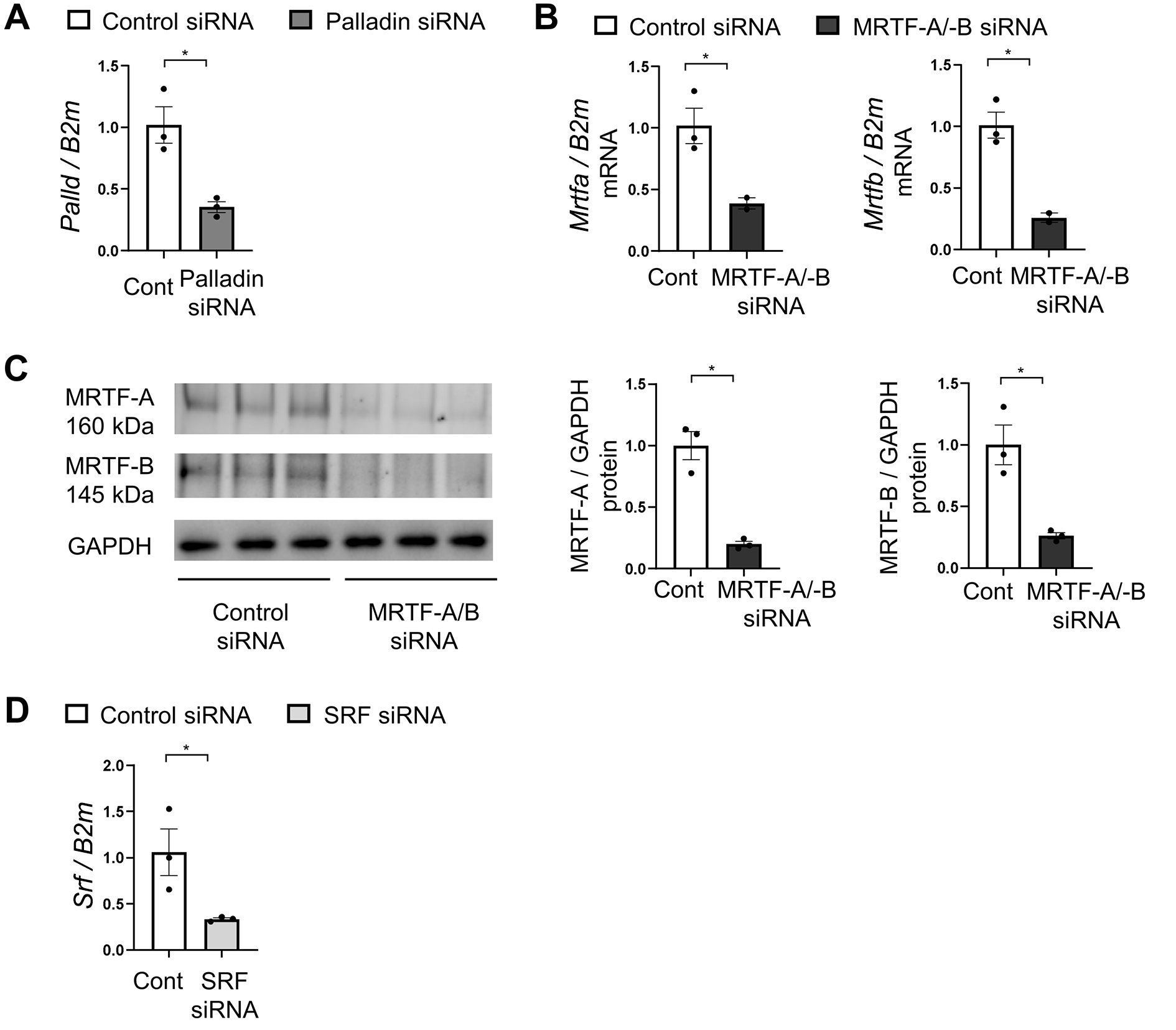


**Figure S4. Validation of siRNA transfection efficiency in renal fibroblasts.** (A) Validation of inhibitory effect of siRNA targeting palladin on *Palld* mRNA expression (*n* = 3 cell preparations/group). (B) Effect of MRTF-A/B knockdown on *Mrtfa* and *Mrtfb* mRNA expression (*n* = 2–3 cell preparations/group). (C) Effect of MRTF-A/B knockdown on MRTF-A/B protein expression (*n* = 3 cell preparations/group). (D) Validation of inhibitory effects of siRNA targeting SRF on *Srf* mRNA expression (*n* = 3 cell preparations/group). The ΔΔCT method was used to calculate the relative expression of target genes and proteins, with β_2_MG (B2m) and GAPDH being the internal control. Mean ± SEM. *Statistically significant.


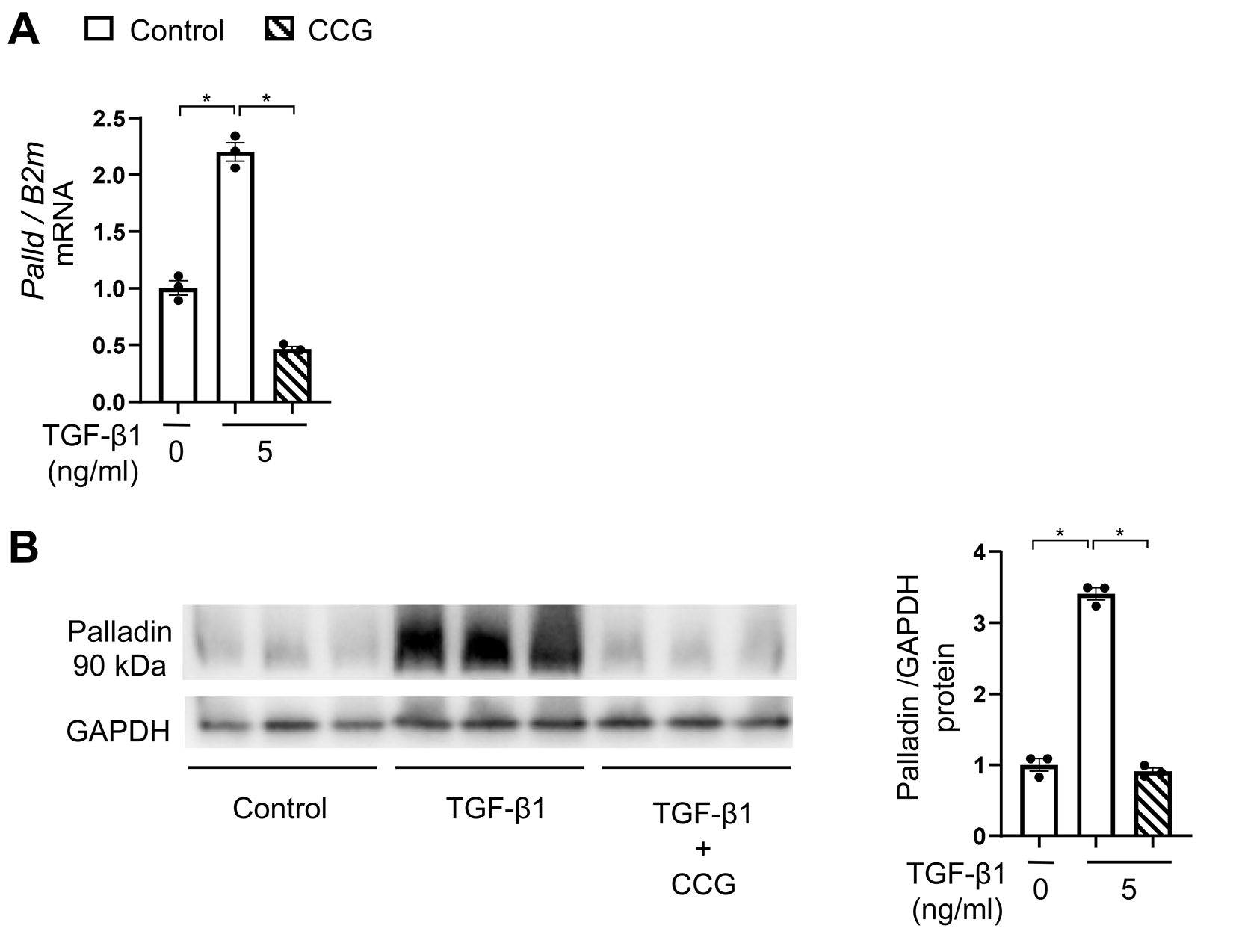


**Figure S5. Palladin expression is suppressed by CCG at mRNA and protein levels.** (A) Effect of pretreatment with CCG-1423 (CCG, 5 μM for 30 min) on *Palld* mRNA expression (*n* = 3 cell preparations/group). (B) Effect of pretreatment with CCG-1423 (CCG, 5 μM for 30 min) on palladin protein expression (*n* = 3 cell preparations/group). The ΔΔCT method was used to calculate the relative expression of the target gene and protein, with β_2_MG (*B2m*) and GAPDH being the internal controls. Mean ± SEM. *Statistically significant.


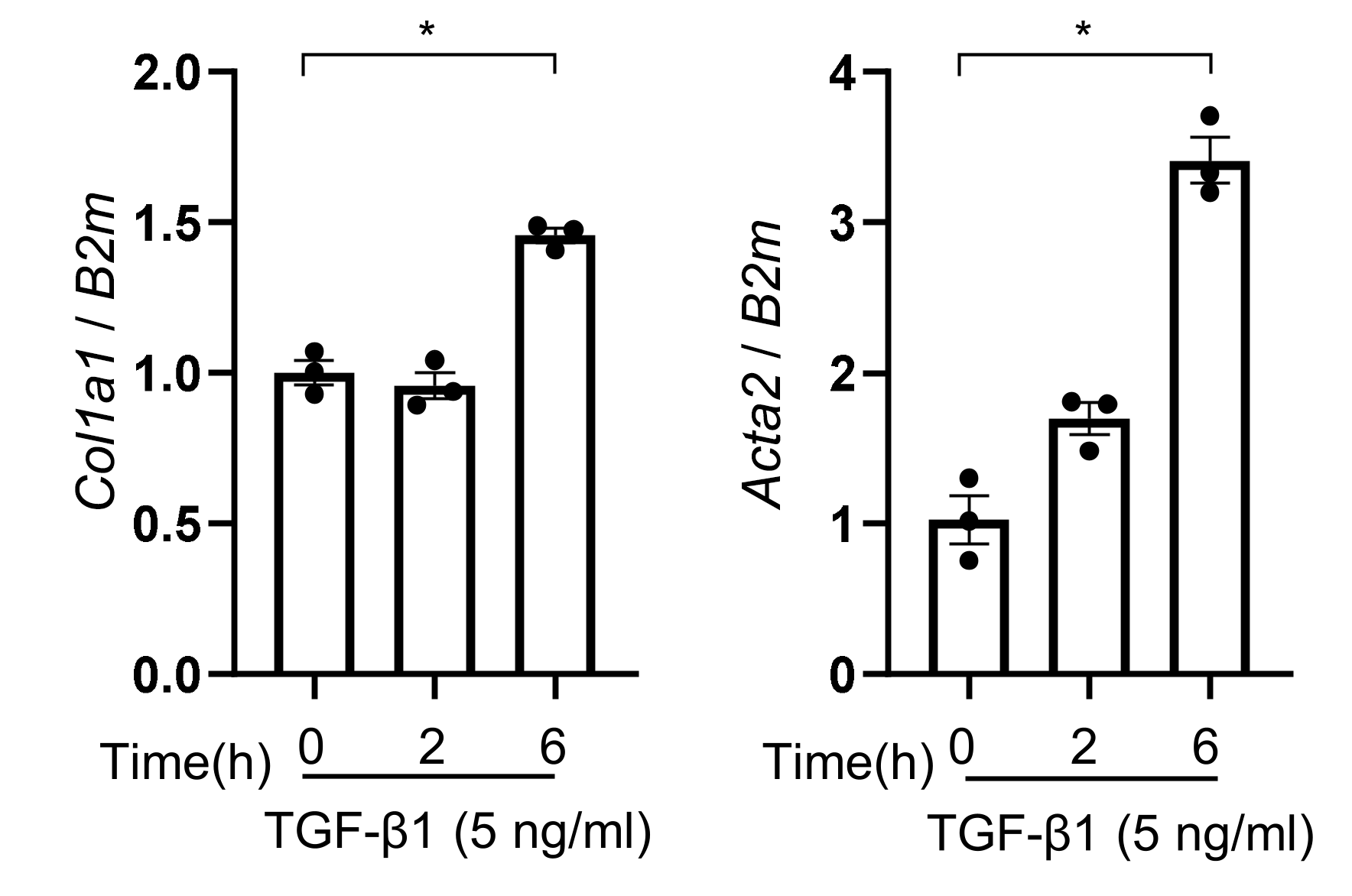


**Figure S6. TGF-β1 enhances expression of *Col1a1* and *Acta2* mRNA in mouse renal fibroblasts.** TGF-β1 induced mRNA expressions of *Col1a1* and *Acta2* in a time-dependent manner in renal fibroblasts (*n* = 3 cell preparations/group). The ΔΔCT method was used to calculate the relative expression of target genes, with β_2_MG (*B2m*) being the internal control. Mean ± SEM. *Statistically significant.

**
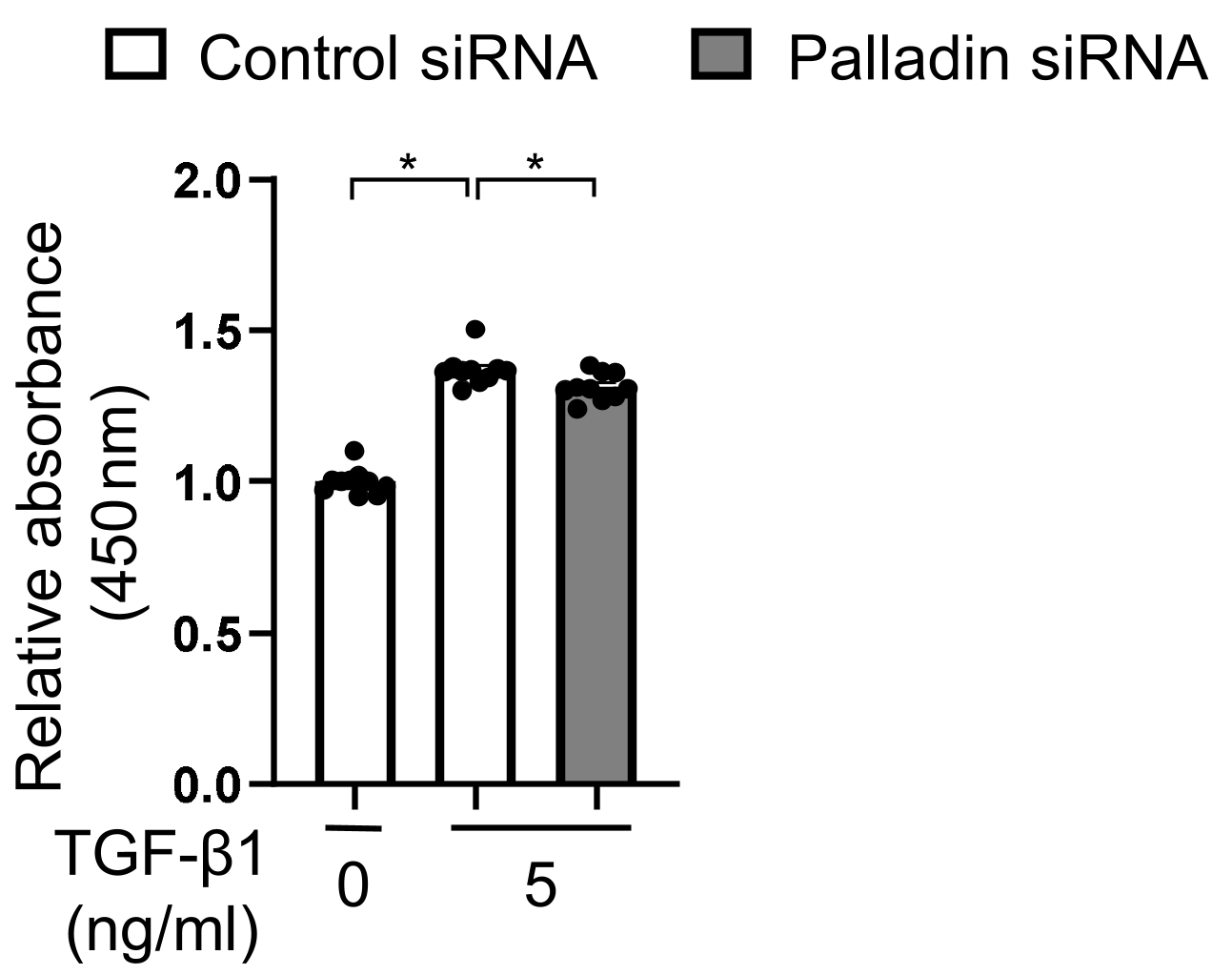
**

**Figure S7. Palladin regulates fibroblast proliferation.** MTT assay: TGF-β1-induced proliferation of renal fibroblasts was suppressed by palladin knockdown (*n* = 10 cell preparations/group).


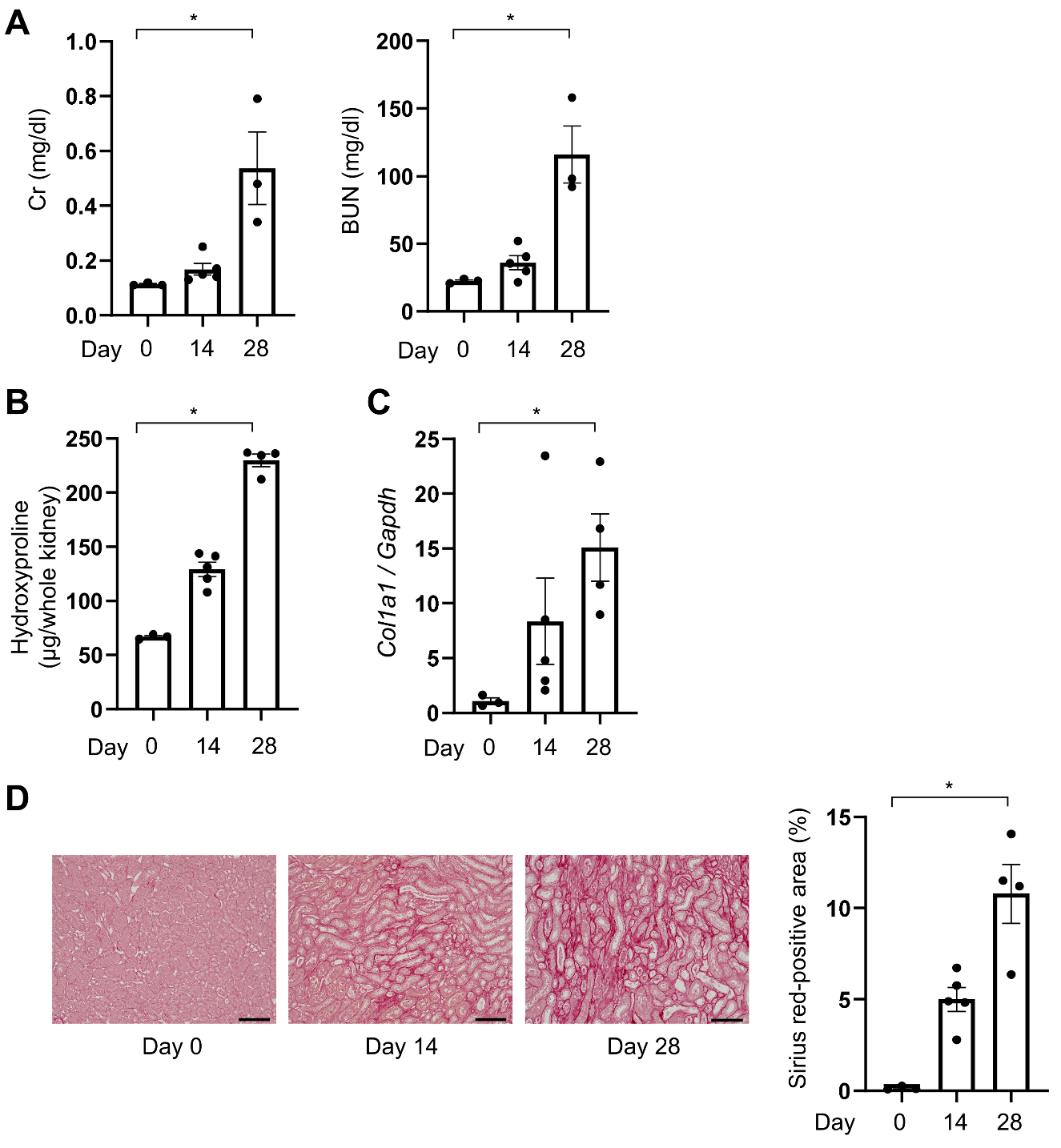


**Figure S8. Adenine administration induces kidney dysfunction and fibrosis in mice.** (A) Serum creatinine and BUN levels were increased in a time-dependent manner in adenine-treated palladin^F/F^ (*n* = 3–5 mice/group). (B) Biochemical analysis of adenine-induced kidney fibrosis: hydroxyproline content increased time-dependently in adenine-treated palladin^F/F^ following adenine administration for 28 days (*n* = 3–5 mice/group). (C) Time-dependent COL1A1 expression in adenine-treated palladin^F/F^ (*n* = 3–5 mice/group). (D) Representative picrosirius red-stained kidney sections of adenine-treated palladin^F/F^ and quantitative analysis of picrosirius red staining in kidney sections (*n* = 3–5 mice/group) for indicated time. Scale bars, 100 µm. The ΔΔCT method was used to calculate the relative expression of target genes, with *Gapdh* being the internal control. Mean ± SEM. *Statistically significant.


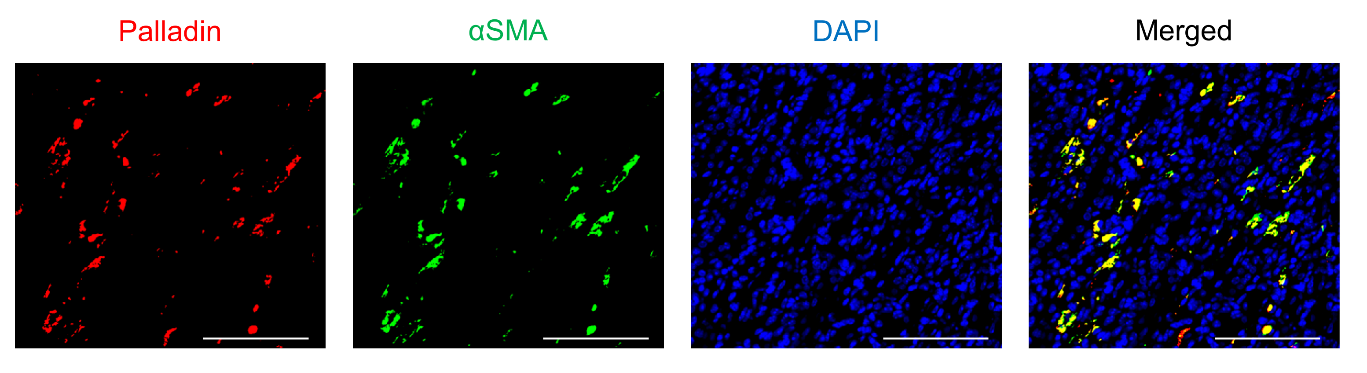


**Figure S9. Palladin colocalizes with αSMA-expressing cells in interstitial space of fibrotic kidney.** Representative kidney sections stained with anti-palladin antibody/anti-αSMA antibody in adenine-treated palladin^F/F^ after adenine administration for 28 days. Scale bars, 100 µm.

**
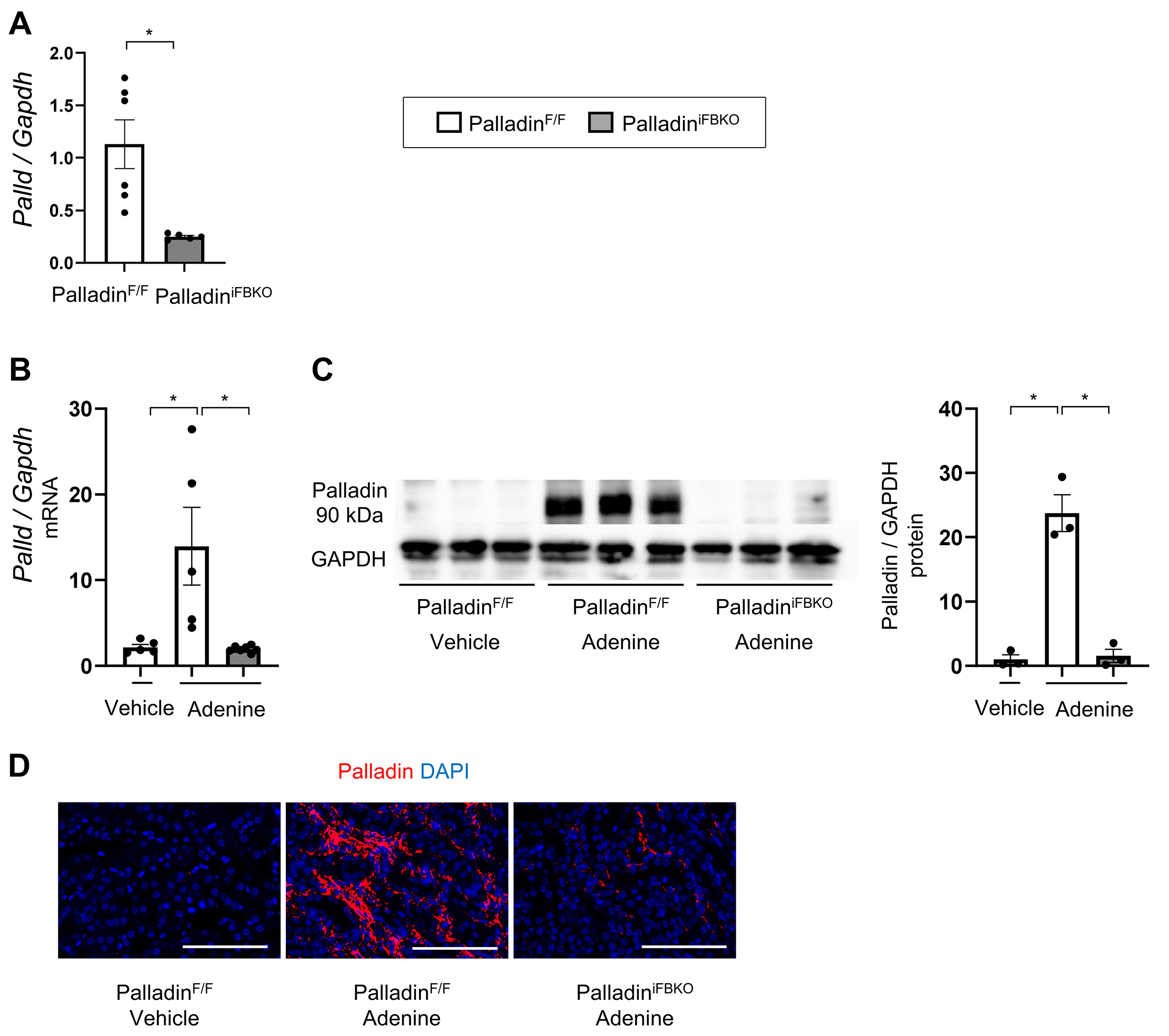
**

**Figure S10.** **Palladin is remarkably suppressed in whole kidney as well as fibroblasts of palladin^iFBKO^.** (A) Palladin expression in dermal fibroblasts obtained 2 weeks after treatment with tamoxifen or vehicle in palladin^iFBKO^. (B,C) Expression of (B) palladin (*Palld*) mRNA and (C) protein in vehicle- or adenine-treated palladin^F/F^ and adenine-treated palladin^iFBKO^ (*n* = 3–5 mice/group). (D) Representative kidney sections stained with anti-palladin. Scale bars, 100 µm. The ΔΔCT method was used to calculate the relative expression of target genes, with *Gapdh* being the internal control. Mean ± SEM. *Statistically significant. The GAPDH panel shown here is the same image as in Figure 5C, because both panels derive from the same sequentially reprobed membrane.


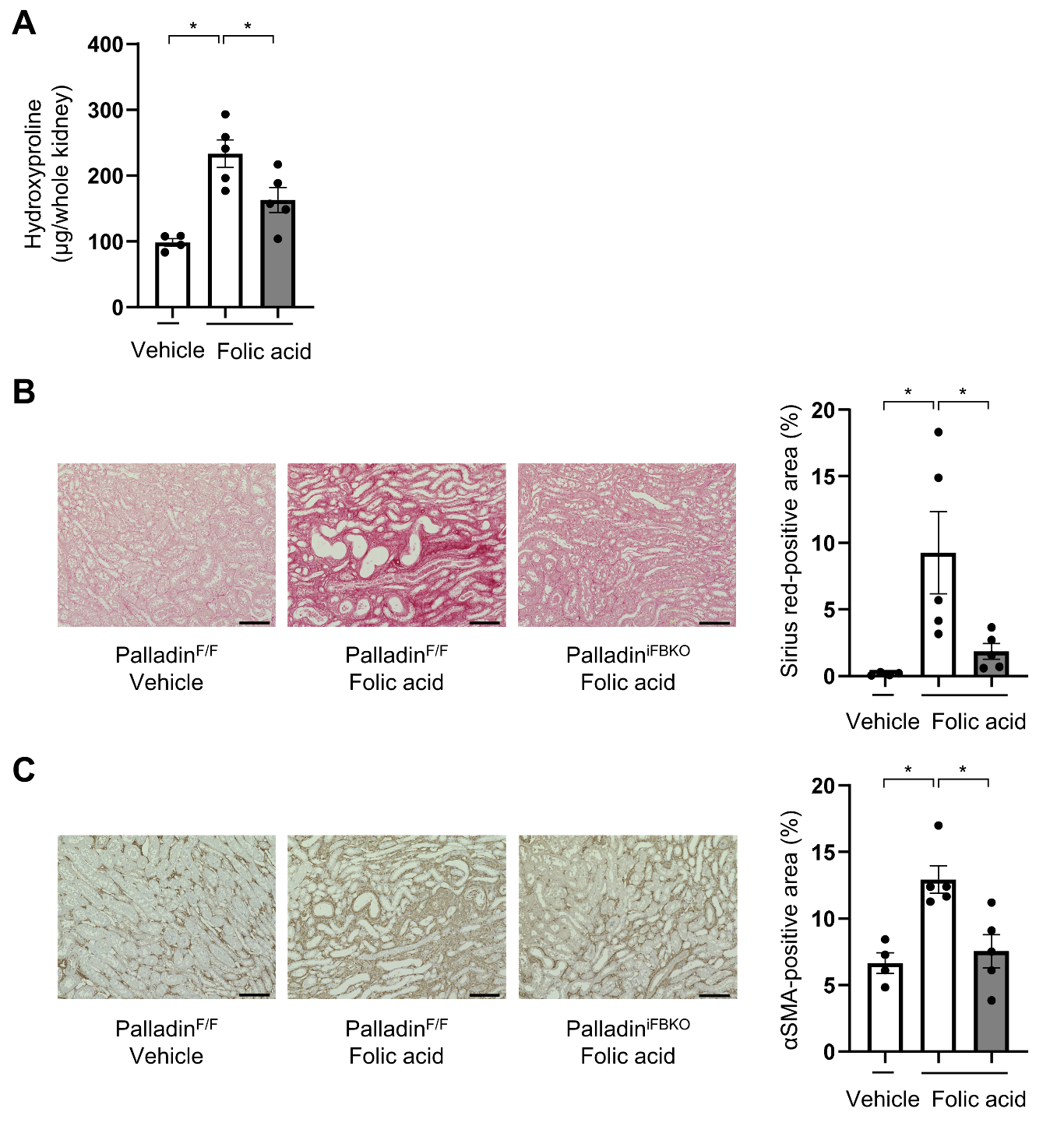


**Figure S11.** Fibroblast-specific palladin deletion ameliorates kidney fibrosis in folic acid-induced nephropathy. (A) Biochemical analysis of folic acid-induced kidney fibrosis: hydroxyproline content was measured in kidney following folic acid administration (*n* = 4 or 5 mice/group). (B,C) Representative images of (B) picrosirius red-stained and (C) αSMA-stained kidney sections of vehicle- or folic acid-treated palladin^F/F^ and folic acid-treated palladin^iFBKO^, with quantitative analysis of each staining in kidney sections (*n* = 4 or 5 mice/group). Scale bars, 100 µm. Mean ± SEM. *Statistically significant.


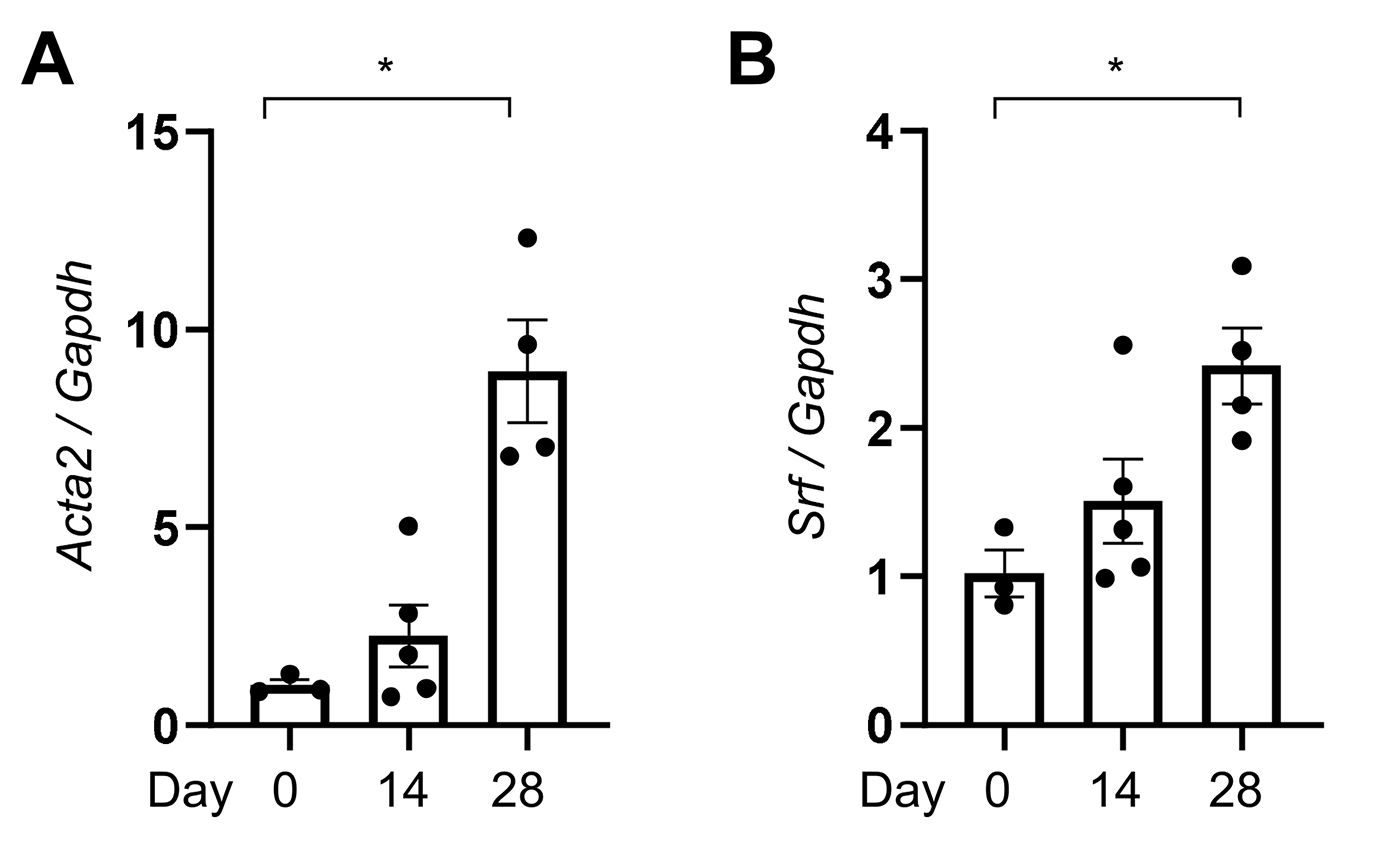


**Figure S12. Adenine administration upregulates expression of *Acta2* and *Srf*.** Time-dependent expressions of *Acta2* and *Srf* in adenine-treated palladin^F/F^ (*n* = 3–5 mice/group). The ΔΔCT method was used to calculate the relative expression of target genes, with *Gapdh* being the internal control. Mean ± SEM. *Statistically significant.


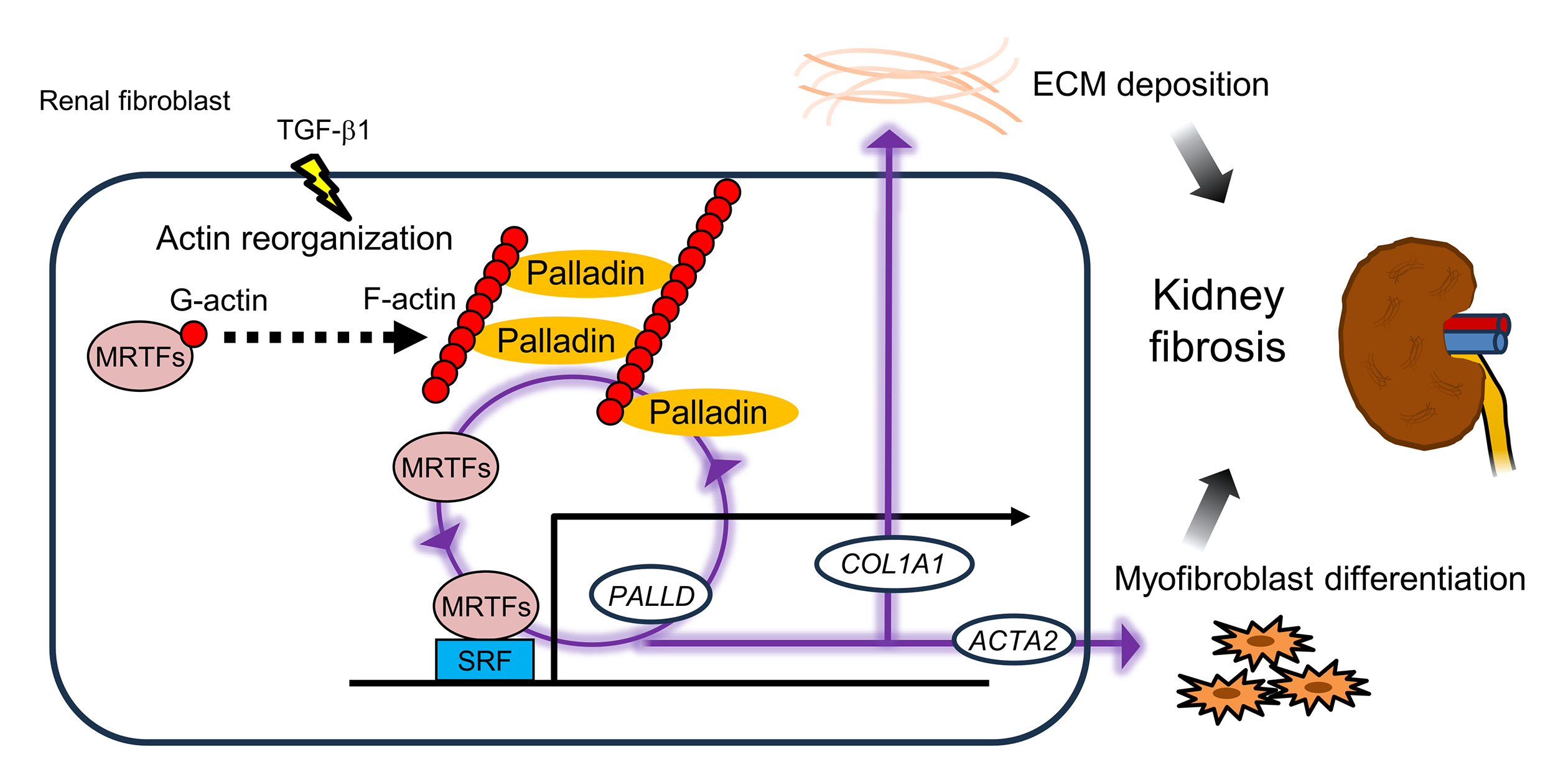


**Figure S13. Proposed schema for profibrotic circuits of palladin–MRTF–SRF axis in pathogenesis of kidney fibrosis.**

**Supplementary Tables S1, S2**

**Table S1.** Primers for reverse transcription-quantitative polymerase chain reaction (RT-qPCR).

Mouse Palladin-F: 5′-TCTGTCCTGCTCTGTTGGTG-3′

Mouse Palladin-R: 5′-GGGGTCCACTCCCTCATTAT-3′

Mouse Vinculin-F: 5′-TCAGACCCATACTCGGTTCC -3′

Mouse Vinculin -R: 5′-AGCCTCATCGAAGGTAAGCA -3′

Mouse Zyxin-F: 5′-TCCATTGAGGCAGATGACAA -3′

Mouse Zyxin-R: 5′-TCAACTCAGGTCTGGGCTCT -3′′

Mouse Cofillin 1-F: 5′-GGCTGTCTCTGATGGTGTCA -3′

Mouse Cofillin 1-R: 5′- GCCTTCTTGCGTTTCTTCAC -3′

Mouse Profilin 1-F: 5′- GGCAAAGACCGGTCAAGTTT -3′

Mouse Profilin 1-R: 5′- GAGTCCCGGATCACAGAACA -3′

Mouse Actinin α1-F: 5′- TGCCTCATCAGCTTGGGTTA -3′

Mouse Actinin α1-R: 5′- ACAATGCTCATGATTCGGGC -3′

Mouse VASP-F: 5′- TCCTTTCCCTTCACCATCCC -3′

Mouse VASP-R: 5′- CCTCCTTCCTTCCCTCACTG -3′

Mouse SRF-F: 5′- ACCTCCACAATCCAGACAGC -3′

Mouse SRF-R: 5′- GGTGCCAGGTAGTTGGTGAT -3′

Mouse MRTF-A-F: 5′- TGAATTGCACTTTGCTCCTG -3′

Mouse MRTF-A-R: 5′- GTCGTGTCCATCCAGGAAGT -3′

Mouse MRTF-B-F: 5′- GCAGTGATGCTTTGTCTCCA -3′

Mouse MRTF-B-R: 5′- GGCTGGTCTGCAGTTAGAGG -3′

Mouse COL1A1-F: 5′-GACTGGCAACCTCAAGAAGG-3′

Mouse COL1A1-R: 5′-TGCTGTAGGTGAAGCGACTG-3′

Mouse ACTA2-F: 5′- CTGACAGAGGCACCACTGAA -3′

Mouse ACTA2-R: 5′- CATCTCCAGAGTCCAGCACA -3′

Mouse β_2_MG-F: 5′-CCGAACATACTGAACTGCTACG-3′

Mouse β_2_MG-R: 5′-CCCGTTCTTCAGCATTTGGA-3′

Mouse GAPDH: Thermo Fisher Scientific (Catalog number 4352339E)

**Table S2.** Primary antibodies for immunohistochemistry, immunocytochemical analyses assay, and western blotting.

Rabbit anti-palladin polyclonal antibody (10853-1-AP, Proteintech)

Mouse anti-αSMA antibody monoclonal antibody (sc-32251, Santa Cruz Biotechnology)

Rabbit anti-αSMA antibody monoclonal antibody (ab124964, Abcam)

Rabbit anti-MRTF-A antibody polyclonal antibody (sc-32909, Santa Cruz Biotechnology)

Rabbit anti-MRTF-B antibody monoclonal antibody (14613, Cell Signaling Technology)
